# Development of an *Agrobacterium*‐delivered CRISPR/Cas9 system for wheat genome editing

**DOI:** 10.1111/pbi.13088

**Published:** 2019-03-12

**Authors:** Zhengzhi Zhang, Lei Hua, Ajay Gupta, David Tricoli, Keith J. Edwards, Bing Yang, Wanlong Li

**Affiliations:** ^1^ Division of Plant Sciences University of Missouri Columbia MO USA; ^2^ Department of Biology and Microbiology South Dakota State University Brookings SD USA; ^3^ Plant Transformation Facility University of California Davis CA USA; ^4^ Functional Genomics University of Bristol Bristol UK; ^5^ Donald Danforth Plant Science Center St. Louis MO USA

**Keywords:** *Agrobacterium* mediation, CRISPR/Cas9, genome editing, grain regulation. mutations, wheat

## Abstract

CRISPR/Cas9 has been widely used for genome editing in many organisms, including important crops like wheat. Despite the tractability in designing CRISPR/Cas9, efficacy in the application of this powerful genome editing tool also depends on DNA delivery methods. In wheat, the biolistics based transformation is the most used method for delivery of the CRISPR/Cas9 complex. Due to the high frequency of gene silencing associated with co‐transferred plasmid backbone and low edit rate in wheat, a large T_0_ transgenic plant population are required for recovery of desired mutations, which poses a bottleneck for many genome editing projects. Here, we report an *Agrobacterium*‐delivered CRISPR/Cas9 system in wheat, which includes a wheat codon optimized Cas9 driven by a maize ubiquitin gene promoter and a guide RNA cassette driven by wheat U6 promoters in a single binary vector. Using this CRISPR/Cas9 system, we have developed 68 edit mutants for four grain‐regulatory genes, *TaCKX2‐1*,* TaGLW7*,* TaGW2,* and *TaGW8*, in T_0_, T_1_, and T_2_ generation plants at an average edit rate of 10% without detecting off‐target mutations in the most Cas9‐active plants. Homozygous mutations can be recovered from a large population in a single generation. Different from most plant species, deletions over 10 bp are the dominant mutation types in wheat. Plants homozygous of 1160‐bp deletion in *TaCKX2‐D1* significantly increased grain number per spikelet. In conclusion, our *Agrobacterium*‐delivered CRISPR/Cas9 system provides an alternative option for wheat genome editing, which requires a small number of transformation events because CRISPR/Cas9 remains active for novel mutations through generations.

## Introduction

Targeted mutagenesis or precise genomic alteration has long been a dream of biologists and biotechnologists. This dream has come true thanks to the rapid development of genome editing technologies in the last decade using engineered nucleases such as zinc finger nucleases (ZFN) (Carroll, 2011), TAL effector nuclease (TALEN) (Cermak *et al*., 2011; Li *et al*., 2011) and Cluster Regularly Interspaced Short Palindromic Repeats (CRISPR)‐associated (Cas) systems (Jinek *et al*., 2012). These systems operate in a similar fashion to create a double strand break (DSB) at a preselected and defined genomic site. The DSB is subsequently repaired by the error‐prone non‐homologous end joining (NHEJ) or by the error‐free homologous recombination (HR) in the presence of a homologous DNA template (Symington and Gautier, 2011). While HR leads to gene correction or replacement, NHEJ causes insertion, deletion and other mutations at the cleavage locus. Engineered ZFN and TALEN nucleases involve selection and assembly of modular DNA binding units and have been successfully applied to plants (Cai *et al*., 2009; Christian *et al*., 2013; Clasen *et al*., 2015; Haun *et al*., 2014; Li *et al*., 2011, 2012; Maeder *et al*., 2008; Mahfouz *et al*., 2011; Shan *et al*., 2013a; Wang *et al*., 2014; Wright *et al*., 2005; Zhang *et al*., 2015). In contrast, the CRISPR/Cas9 system consists of a short guide RNA (sgRNA) and a conditional DNA nuclease Cas9 (Jinek *et al*., 2012). The 20 nucleotides (nt) at the 5′ end of the sgRNA guide the Cas9/sgRNA complex to search for the target sequence along the chromosomal DNA until an exact match is found. If an NGG trinucleotide, the protospacer adjacent motif (PAM), is located immediately downstream of the target site, Cas9 undergoes a conformational change, which activates two separate nuclease domains in Cas9 and leads to cleavage of the target (Jinek *et al*., 2014). Since the first success in editing genes in cultured human cell lines (Cong *et al*., 2013; Mali *et al*., 2013), application of the CRISPR/Cas9 system has been rapidly expanded into plants and ushered a flood of publication in last several years (reviewed in references: Soyars *et al*., 2018; Weeks *et al*., 2016). The predominant use of CRISPR/Cas9 technology in plants is to target genes of interest for knockout. More recently, the CRISPR system was engineered by Cas9 nickase fused with cytidine deaminase or adenosine deaminase for highly precise base editing (Gaudelli *et al*., 2017; Komor *et al*., 2016; Nishida *et al*., 2016), which has also been adopted in plants. With these features, the engineered CRISPR systems have been playing an important role in the basic understanding of plant biology and crop improvement.

Common wheat, bread wheat, or hexaploid wheat (*Triticum aestivum* L., genomes AABBDD) is the most widely cultivated crop. It provides ~20% of our daily diets and thus plays an important role in global food security and rural economy. To meet the demand for the increasing population, we must increase wheat yield by 50% by the year 2034 (http://iwyp.org/). In this respect, the CRISPR technology is expected to contribute greatly to creating novel variations in wheat. Wheat was one of the plant species first modified by CRISPR/Cas9 (Shan *et al*., 2013b), and several traits have been targeted for mutations, including powdery mildew resistance (Wang *et al*., 2014), grain size (Wang *et al*., 2018; Zhang *et al*., 2018b), and grain quality (Liang *et al*., 2017; Sánchez‐León *et al*., 2018; Zhang *et al*., 2018b). In all these researches, the Cas9 and sgRNA transgenes were delivered by the biolistic bombardment. In recent years, the efficiency of the *Agrobacterium*‐mediated transformation has been improved significantly in wheat (Ishida *et al*., 2015; Richardson *et al*., 2014; Wang *et al*., 2017a). Compared to the biolistics approach, the improved *Agrobacterium*‐mediation systems increased transformation efficiency and expanded the transformability of wheat genotypes. These improvements have not been integrated into CRISPR/Cas9‐based genome editing or other genome editing platforms in wheat. During the preparation of this manuscript, a report was published on *Agrobacterium*‐delivered CRISPR/Cas9 for wheat gene editing in which one gene (*DA1*) was tested in T_0_ transgenic plants with a high frequency of not yet proven inheritable mutations (Zhang *et al*., 2018a). In the present research, we developed an *Agrobacterium*‐delivered CRISPR/Cas9 system for genome editing in wheat and applied it to target four grain‐size regulatory candidate genes. Our result showed that a small number of transformation events are enough for recovering desired mutations in T_0_, T_1_, and T_2_ plants attributed to the continuing activity of CRISPR/Cas9 transgene through generations.

## Results

### Development of a CRISPR/Cas9 system for wheat genome editing

**Figure 1:**
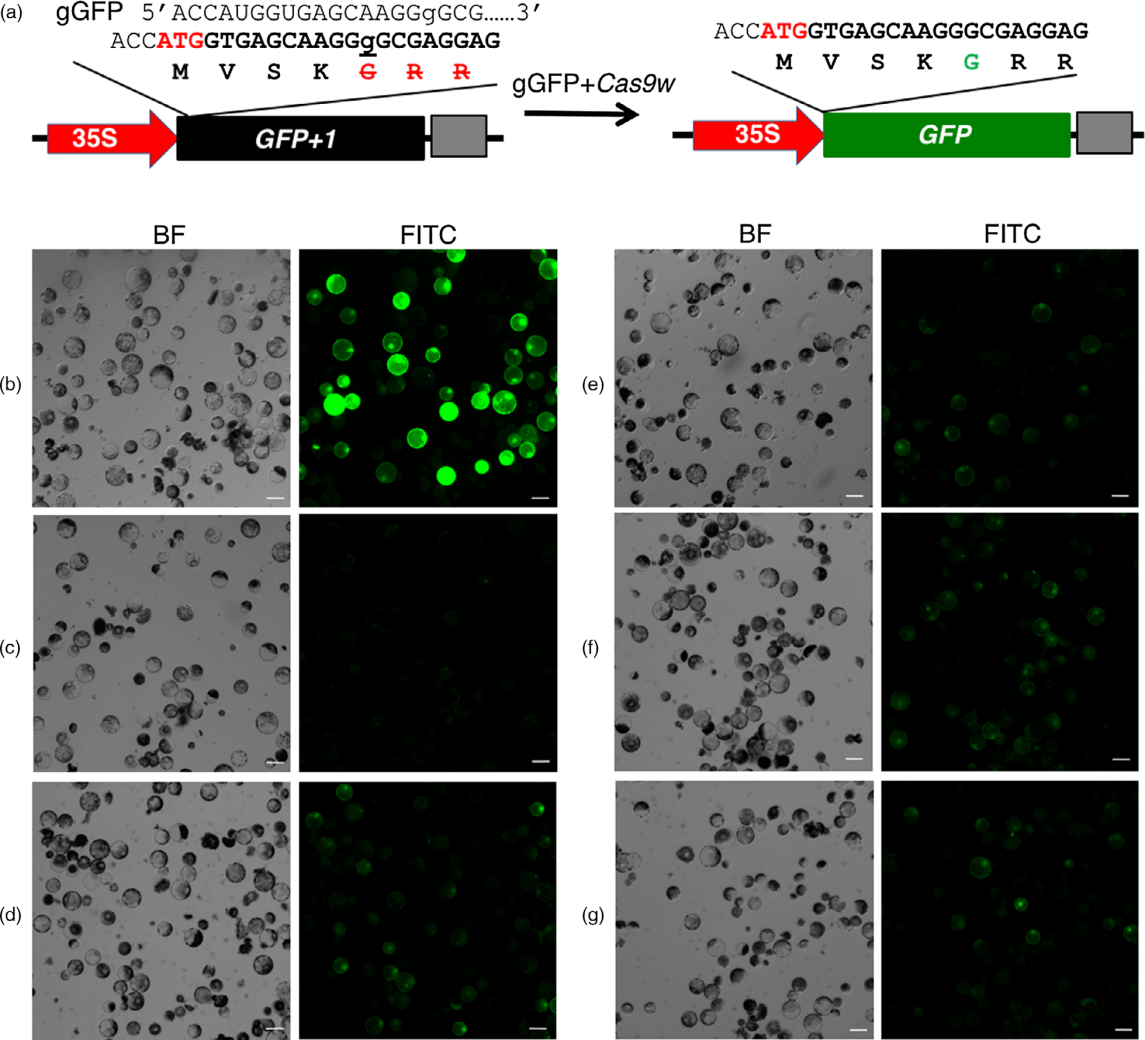
Expression of GFP in protoplasts transfected with *Cas9,* sgGFP, and non‐functional GFP. (a) Strategy to test the wheat CRISPR‐Cas9 system in protoplasts. (b). pOsUbi‐GFP; (c) pUC‐35S:GFP+1; (d) pTaU6.1‐gGFP; (e) pTaU6.2‐gGFP; (f) pTaU6.3‐gGFP; (g) pTaU6.5‐gGFP. BF: Bright field; FITC: Fluorescein isothiocyanate. Scale bars indicate 50 μm.

We first sought for strong U6 promoters from wheat genome to express guide RNA genes. Promoters from four wheat U6 RNA coding genes, i.e., *TaU6.1*,* TaU6.2*,* TaU6.3,* and *TaU6.5* (Appendix S1), were tested individually for their abilities to express the guide RNA gene *gGFP*, which targets the 1‐bp insertion site in the mutated *GFP* gene in the pUC‐35S:GFP+1 for restoration of GFP, together with wheat codon‐optimized *Cas9* in wheat protoplasts (Figure 1a). To do so, we first evaluated the transfection rate of wheat protoplasts using a pOsUbi‐GFP construct as a control. On average, 52.8% of protoplasts were successfully transfected by pOsUbi‐GFP (Figure 1b). When transfected with the pUC‐35S:GFP+1 construct, in contrast, no GFP signal was observed in the protoplasts (Figure 1c), indicating that the 1‐bp frameshift insertion completely abolished the GFP‐coding capacity. When transfected together with the U6 promoter‐driven guide RNA gene *gGFP* and the ZmUbi‐*Cas9*, the GFP signal was detected in the protoplasts (Figure 1d–g). After normalization with the transfection rate from pOsUbi‐GFP, the desired editing occurred at 47.4%–68.5% in four U6 promoters tested (Figure S1). Among them, TaU6.3 showed significantly higher editing rate (68.5% ± 7.9%) than the rest three U6 promoters (*P *<* *0.01). TaU6.1 ranked second in editing efficiency, but it was not significantly different from TaU6.2 (*P *>* *0.08) and TaU6.5 (*P *=* *0.28). In term of average GFP fluorescence intensity per cell, TaU6.3 also showed the best efficiency among the promoters tested (*P *<* *0.05). This result indicated that the Cas9 functions properly in wheat cells, and TaU6.1 and TaU6.3 are best candidates for expressing guide RNAs in wheat. Thus, TaU6.1 was used to drive the sgRNA1 inserted into the *Btg*ZI sites and TaU6.3 to drive the sgRNA2 inserted into the *Bsa*I sites in pTagRNA4 (Figure S2).

To establish an *Agrobacterium*‐based wheat CRISPR/Cas9 system, we chose a binary vector pLC41 (Japan Tobacco, Tokyo, Japan) that was modified as a Gateway recipient vector. A pENTR derived intermediate vector was constructed to express Cas9 under the strong maize ubiquitin gene promoter and for the construction of guide RNA genes under U6 promoters (Figure S3).

### Identification of grain regulatory genes and design of guide RNAs

**Figure 2:**
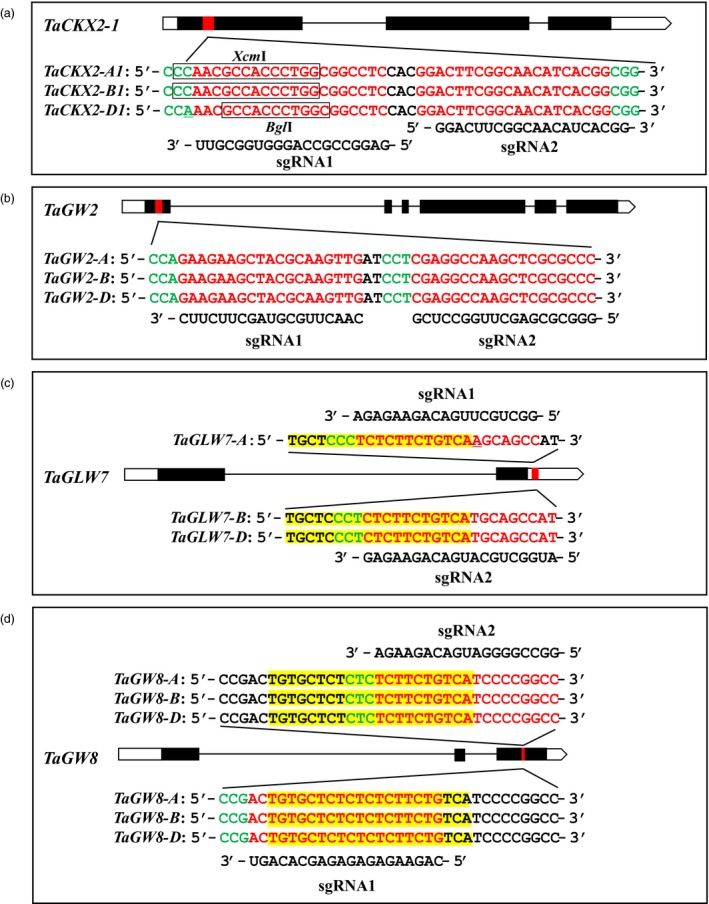
Schematics of gene structures with exons (black bars), introns (black lines) and sequences of target sites and guide RNAs. (a and b) Structures of *TaCKX2‐1* and *TaGW2* with target sites in exon 1. Nucleotides in red and green indicate target sites and PAM sequences, respectively. Underlined nucleotides represent the single nucleotide polymorphism (SNP) in three homeologues. Boxed letters indicate the restriction enzyme site. The solid red bars are the conserved region of the target genes. (c) Structures of *TaGLW7* with target sites located in 3′ UTR and (d) structures of *TaGW8* with the target sites in exon 3. Highlighted yellow regions indicate microRNA recognition sites.

**Figure 3:**
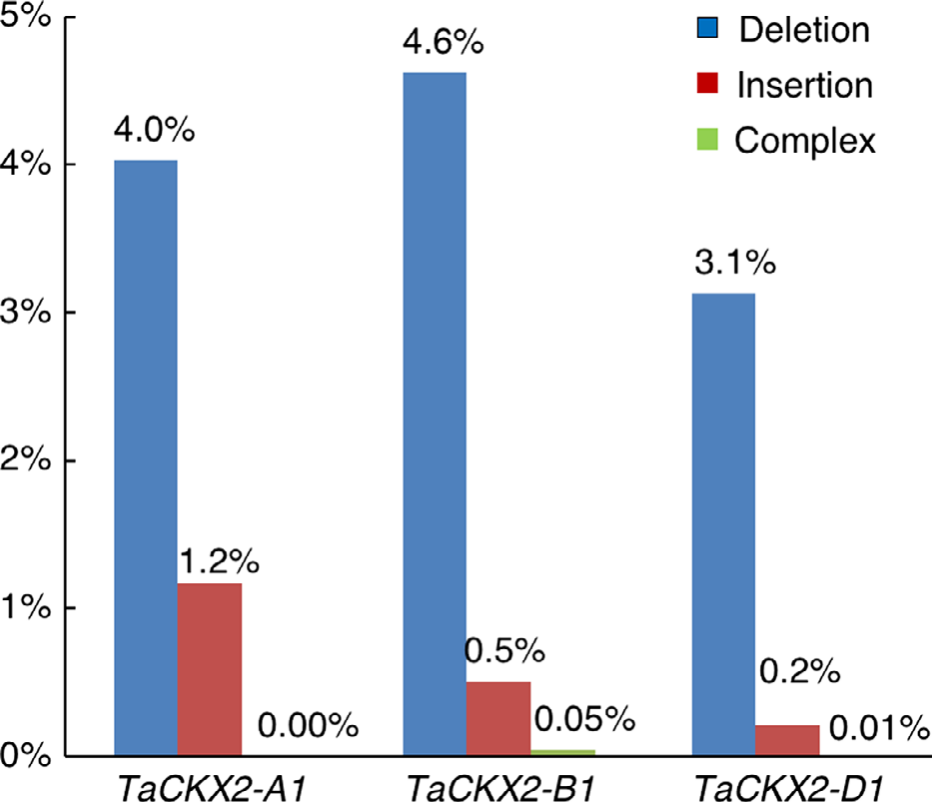
Types and frequencies of mutation in T_0_ plants discovered by PCR amplicon deep sequencing.

In a previous study, we identified 45 orthologus loci as candidate genes for wheat grain regulatory genes, 23 of which are negative regulators or expression is negatively regulated by microRNAs (Li and Yang, 2017). From these 23 genes, we selected two negative regulators *TaCKX2‐1* and *TaGW2*, and two microRNA‐regulated positive regulators *TaGLW7* and *TaGW8* for the present research, two guide RNAs were designed for each gene to target the sequences conserved among the A, B and D genomes of the hexaploid wheat Fielder (Table S1). For *TaCKX2‐1* and *TaGW2*, guide RNA sequences are located in a conserved region of their first exon, and perfectly matched their targets. An SNP was found in the first position of PAM motif (5′‐NGG‐3′) in *TaCKX2‐D1* (Figures 2a and 3b), it should not affect the editing efficiency because the nucleotide at that position is degenerate. For *TaGLW7* and *TaGW8*, the guide RNAs target the microRNA recognition sites (Figure 2c,d), the two guide RNA target sequences are overlapped on two strands. For *TaGLW7* gene, a single nucleotide mismatch is found in the guide RNA target sequences among the homoeologous genes. Accordingly, two guide RNA genes were designed with sgRNA1 targeting the *TAGLW7‐A* and sgRNA2 targeting *TaGLW7‐B* and *TaGLW7‐D* (Figure 2c).

### *Agrobacterium*‐delivery of CRISPR/Cas9 system into wheat cells

Four CRISPR constructs were individually introduced into Fielder immature embryos through *Agrobacterium*‐mediated gene transfer. After selection and regeneration, eight plants for *TaCKX2‐1*, four for *TaGW2*, two for *TaGLW7*, and eight plants for *TaGW8* were obtained. Plants grow normally in growth chamber or greenhouse without obvious morphological alteration. PCR screening with Cas9 and sgRNA specific primers identified that all plants except one from *TaCKX2‐1*, contain both Cas9 and sgRNA (Table S2). Gene‐specific primers flanking the target region were used successfully to PCR‐amplify the expected fragments from all three loci of each gene. However, a T7 endonuclease 1 (T7E1) assay with PCR products did not reveal the expected digested pattern for the InDel (insertion/deletion) mutation. Sanger sequencing of the PCR products confirmed that all examined plants carry only the wild type allele for *TaCKX2‐1, TaGW2*,* TaGLW7,* and *TaGW8*.

### Detection of mutations in T_0_ plants by deep sequencing

We were not able to detect a mutation in T_0_ plants by T7E1 assay and Sanger sequencing directly on PCR products. This result could be due to low edit frequency in wheat as shown in prior studies (Wang *et al*., 2014, 2018), but somatic mutations may have occurred in the T_0_ plants. To test this hypothesis, we sequenced the PCR products derived from the *TaCKX2‐1* target sites by the HiSeq 2000 platform. A total 1 101 752 raw reads were obtained from the deep sequencing, of which 486 890 reads meet the quality standards. Approximately 48% and 47% of the quality reads were mapped to the *TaCKX2‐B1* and *TaCKX2‐D1*, respectively, but only 0.2% of them were mapped to the *TaCKX2‐A1*. Both *TaCKX2‐A1* and *TaCKX2‐B1* showed 5.2% of mutation rate, while *TaCKX2‐D1* had a lower mutation frequency of 3.3% (Figure 3). Deletions occurred at much higher frequencies than insertions in all three homoeologous genes. Deletion frequency is 4.0%, 4.6%, and 3.1% for *TaCKX2‐A1, TaCKX2‐B1* and *TaCKX2‐D1*, respectively. By contrast, insertion frequency at these loci are 1.2%, 0.5%, and 0.2%, respectively. There are also rare cases of complex editing (simultaneous deletion and insertion) in *TaCKX2‐B1* and *TaCKX2‐D1*, but they all happened at <0.05% (Figure 3).

**Figure 4:**
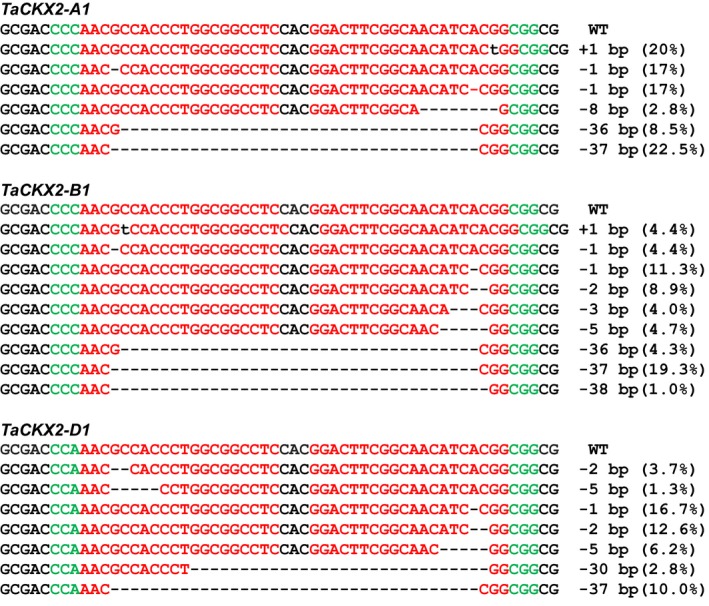
Representative InDel mutations in *TaCKX2‐1* from T_0_ transgenic plants. Green letters indicate the PAM sequences. The dashed lines represent nucleotide deletions. Insertion is shown by lower case letters in black. The number in the parenthesis represents the frequency of the mutation type in the total mutations detected.

The lengths of the deletion range from 1 to 37 bp in *TaCKX2‐A1*, 1 to 47 bp in *TaCKX2‐B1*, and 1 to 72 bp in *TaCKX2‐D1*. Interestingly, a 37‐bp deletion from position 136 to position 172 downstream of the translation start site (ATG) occurs at a high frequency. It is the most abundant mutant allele in *TaCKX2‐A1* and *TaCKX2‐B1*, with 22.5% and 19.3% of the total mutations, respectively. This deletion, occurred at 10% in *TaCKX2‐D1*, is the third most abundant mutation next to the 1‐bp deletion at position 165 (16.7%) and 2‐bp deletion at positions 172 and 173 (12.6%). In *TaCKX2‐A1* and *TaCKX2‐B1*, the 1‐bp and 2‐bp deletions at 3 bp upstream of the PAM sites of two sgRNAs rank in abundance just next to the 37‐bp deletion (Figure 4). Overall, the deletion frequency at the sgRNA2 target site is higher than that at the sgRNA1 target site (Figure S4), suggesting that TaU6.3 may be more active in transcription of sgRNA, or sgRNA2 for *TaCKX2‐1* had the better capability in directing the Cas9‐sgRNA binding to the target region. These results showed that our CRISPR/Cas9 system did work in the T_0_ transgenic plants.

### Screening mutations in T_1_ generation

Considering that the CRISPR/Cas9 system functions in the somatic cells of the T_0_ transgenic plants, we expected mutations would also occur in gametes and embryonic cells, and screened T_1_ progenies carrying the *Cas9* transgene for targeted mutations. To investigate CRISPR/Cas9 induced mutation at the sgRNA target sites, we used three gene‐specific primer pairs to amplify each homoeologue, which contained the genomic region of two sgRNA target sites, and Sanger sequencing of PCR amplicons for all T1 plants was performed. The PCR restriction enzyme digestion assay (PCR‐RE) and T7E1 cleavage assay were not conducted for independent T1 progenies, as only one restriction site existed in the target sequence of TaCKX2‐1 sgRNA1, while T7E1 assay sometime failed to screen homozygous mutants and heterozygous mutants with small deletion or insertion mutation. Twenty‐five plants containing 10 types of mutations were identified for four target genes from 226 T1 plants (Tables S3 and S5).

**Figure 5:**
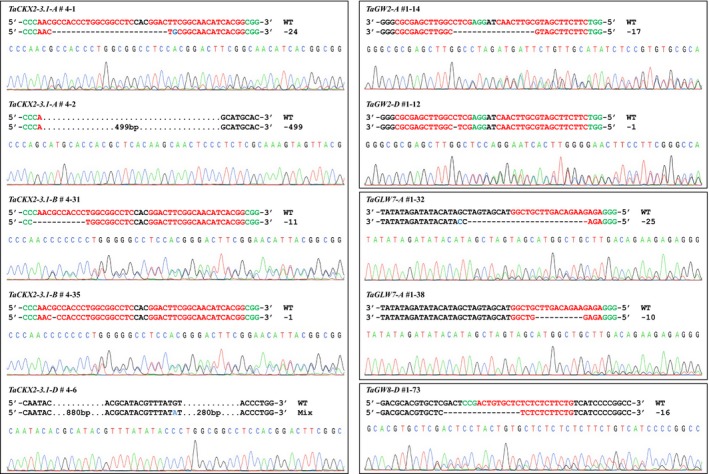
CRISPR/Cas9 induced mutations in target genes. Sequencing chromatograms of PCR amplicons from the selected T_1_ mutants and corresponding regions are shown in (a–d) *TaCKX2‐1*,* TaGW2*,* TaGLW7,* and *TaGW8*. The PAM sequences and target sequences are highlighted in green and red, respectively. The dashes lines indicate deletion, and the blue letters represent substitution. Black dots indicate nucleotides not shown.

For *TaCKX2‐1* gene, we screened three T_1_ populations derived from T_0_ plants #1, #2, and #4. From the 44 T_1_ plants of the #4 family, we detected two mutations in *TaCKX2‐A1* and two mutations in *TaCKX2‐B1*. A 24‐bp deletion and 1‐bp substitution (#4‐1) and a 499‐bp deletion (#4‐2) were found in *TaCKX2‐A1* (Figure 5a; Figure S6b), and a 11‐bp deletion (#4‐31) and 1‐bp deletion (#4‐35) were identified in *TaCKX2‐B1* (Figure 5a; Figure S6c). Two plants #4‐31 and #4‐35 showed no PCR amplification in *TaCKX2‐D1*, while PCR amplicons were amplified in both *TaCKX2‐A1* and *TaCKX2‐B1* (Figures S6b, S6c, and S6d). Using a long‐range PCR primer pair, we amplified a product of ~2.5 kb in the wild type and ~1.3 kb in homozygous mutant plants (Figure S6d), suggesting that ~1.2 kb deletion occurred in *TaCKX2‐D1*. As the ~2.5 kb band is weak in wild type and heterozygous plants (Figure S6d), a new primer pair were designed with the PCR products size being ~1.7 kb to screen the 44 *Cas9*‐positive plants in the #4 T_1_ family (Figure S5a,b). The PCR and Sanger sequencing results showed that 16 mutant plants carry an identical mutation, a complex mutation with deletion of 1160 bp in *TaCKX2‐D1* (Figure S7), of which three are homozygous, and 13 are heterozygous (Table S5), indicating the deletion occurred in T_0_ and transmitted to T_1_ generation. It was not detected by deep sequencing because the deletion was much larger than the insert size of the sequencing library. Thus, the edit rate in the T_0_ plants is 12.5% (1 in 8 T_0_ plants) for *TaCKX2‐D1*. Four new mutations were detected in *TaCKX2‐A1* and *TaCKX2‐B1*, each of which contained two heterozygous deletion mutants (Figure S6; Table S3). Thus, mutagenesis frequencies of the *TaCKX2‐A1* and *TaCKX2‐B1* were 4.5% (2/44) in the T_1_ generation.

For *TaGW2*, a 1‐bp deletion in *TaGW2‐A*, and a 17‐bp deletion in *TaGW2‐D* were detected in 15 *Cas9*‐positive T_1_ plants with an edit efficiency of 13.3% (Table S3; Figure 5b; Figure S5c). For *TaGLW7*, we found two mutants from 74 T_1_ plants derived from two T_0_ plants with an edit efficiency of 2.7%, and both mutations were deletions within the microRNA recognition site of *TaGLW7‐A* (Figure 5c). Of 93 *TaGW8* T1 plants, one mutant with a 16‐bp deletion was identified (Figure 5d), with an edit frequency of 1.1%. Taken together, edit efficiency in T_1_ progeny varied among the targets. Of the 25 mutated plants among four target genes, ten types of mutations were detected, and all of them were deletions ranging from 1 to 1160 bp (Figure 5a). Because both target sites and gene‐specific PCR primer binding sites were within the region of the 1160‐bp deletion (complex edit with 880‐ and 280‐bp deletions and a 1‐bp substitution) (Table S4) in *TaCKX2‐D1*, it is possible that the mutated allele was initially missed with the primers in the T_0_ plant #4.

We also screen T_1_ populations for transgene segregation. Transgenes segregated into 15 (present) to 1 (absent) in *TaGLW7*_T1‐32 and _T1‐38 families (*P *>* *0.55698) and into 63 (present) to 1 (absent) in *TaGW2* T1‐12 family (*P *=* *0.824132), indicating two and three copies of transgenes in the T_0_ transgenic plants, respectively. From these populations, we identified two transgene‐free mutants, one for *TaGLW7‐A* and one for *TaGW2‐D*.

### Inheritance of induced mutations in T_2_ progenies

To confirm inheritance of *TaCKX2‐1* mutations in T_2_ generation, two T_1_ lines, #4‐1 that contained a 24‐bp deletion and a 1‐bp substitution mutation in *TaCKX2‐A1*, and #4‐35 that carried a 1‐bp deletion in *TaCKX2‐B1*, were allowed to self‐pollinate for generation of T2 populations. Totally, 24 T_2_ individuals were *Cas9*‐positive and carried mutations at the *TaCKX2‐A1* and *TaCKX2‐B1*. Of 17 T_2_ plants from #4‐1, two were homozygous mutants, eight heterozygous mutants and seven wild type plants (Figure S8a), while two homozygous mutants, four heterozygous mutants, and one wild type plant were identified from the T_2_ plants of #4‐35 by using PCR‐RE assay (Figure S8b). The segregation ratios fit the 1:2:1 expectation (*P *>* *0.22313). Sanger sequencing results showed that all the T_2_ mutation types in the *TaCKX2‐A1* and *TaCKX2‐B1* were consistent with their T_1_ mutations. We screened 48 T_2_ progenies from the T_1_ mutants for *TaGW2‐A* and *TaGW2‐D*. Their segregation also followed a 1:2:1 ratio (*P *>* *0.14370). These results indicate that the CRISPR/Cas9 induced mutations are heritable in wheat.

### Edit mutations in the T_2_ and T_3_ generations

**Figure 6:**
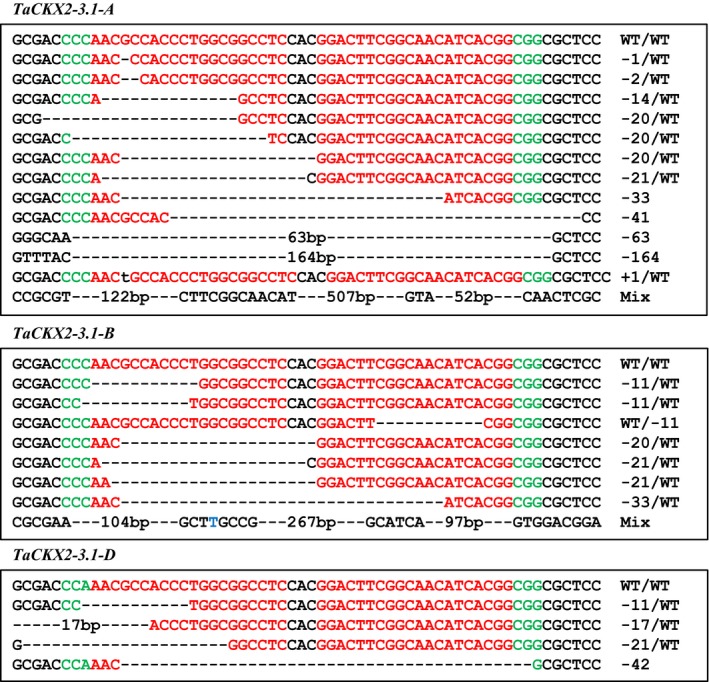
Sequence information of new *TaCKX2‐A1*,* TaCKX2‐B1* and *TaCKX2‐D1* mutations in the T_2_ progeny plants from event #4‐6. The mutation types include deletions (dashed line), insertions (lower‐case letters in black) and substitutions (blue letters). PAM sequences are in green.

We found one new mutation in the T_2_ progenies of #4‐1 (#4‐1‐9), which was due to a complex deletion of 218, 18 and 13 bp at the *TaCKX2‐D1* locus. No additional mutation was detected in the T_2_ progenies of line #4‐35 due to small population size. To further investigate whether new mutations were induced by CRISPR/Cas9 system in the T_2_ generation, we screened progenies of three *Cas9*‐positive T_1_ plants, #4‐6, #4‐10, and #4‐18 for analysis of *TaCKX2‐1* targets because they produced more seeds than other plants. The T_1_ plant #4‐6 is heterozygous for the 1160‐bp deletion in *TaCKX2‐D1* and is wild type in *TaCKX2‐A1* and *TaCKX‐B1* (Figure S7). A total of 180 T_2_ plants from line #4‐6 were screened using PCR‐RE (Figure S9). Fifty‐one new mutations were identified including 27 in *TaCKX2‐A1*, 18 in *TaCKX2‐B1*, and 6 in *TaCKX2‐D1* homoeologous loci in addition to the 1160‐bp deletion in *TaCKX2‐D1* (Figure 6; Table S4). While most of the newly identified mutations were heterozygous, two mutations, 1‐bp deletion in #4‐6‐226 and 2‐bp deletion in #4‐6‐47 in *TaCKX2‐A1* (Figure S9a; Table S6), are homozygous. Because no heterozygotes were found in the T_2_ population, the homozygous mutations were most likely derived from fertilization of the two independent deletions at the same positions from the egg and sperm cells. From T_2_ and T_3_ populations, we recovered 35 homozygous double mutants (mutations in two of the three homoeologous genes) and 33 triple mutants (mutations in all three homeologous genes), ten of which are homozygous in two homoeologous loci of *TaCKX2‐1* (Table S7).

We also screened 46 T_2_ plants from #1‐32 line for *TaGLW7* mutations using PCR‐RNP assay (Figure S10) and found novel mutations in the *TaGLW7‐A* and *TaGLW7‐D* gene. Two independent mutant plants carried the same mutation type of 1‐bp insertion in *TaGLW7‐A,* and a novel 5‐bp deletion was found in *TaGLW7‐D*. The results suggest that the occurrence of novel mutations may be associated with the continuing activity of the Cas9/gRNA complex.

### No off‐target mutation detected via Sanger sequencing of PCR‐amplicons in a CRISPR active population

We next assessed the potential off‐target effect by the wheat CRISPR/Cas9 system. Considering that Cas9 and the guide RNA for *TaCKX2‐1* were most active to induce on‐target mutations in the #4‐6 T_2_ family (i.e., 51 new mutations in 180 plants), we selected progeny, 24 *TaCKX2‐1* mutants and 24 wild type plants, of this family to assess the off‐target effect by this most active gRNA. The *TaCKX2‐1* target sequences plus the PAM motifs were used to search the wheat reference genome, resulting in eight potential off‐target sites with 2 and 3 bp mismatches compared to the target sequences. Two of the eight off‐targets are unique in the wheat genome, while the remaining six contain two sites, each of which are triplicated in the wheat genome. We selected the top four of eight loci to design gene and allele specific primers for PCR amplification of relevant regions (Table S8). Individual PCR amplicons (*n* = 192) were subjected to Sanger sequencing, and the sequencing chromatograms were manually analysed for any sign of double tracing peaks at the target regions. The results showed that no mutation occurred at these four off‐target sites via the targeted Sanger sequencing method. However, it is possible that unintended mutations occur in some somatic cells, rare mutations that can be detected only by using more sensitive methods such as deep sequencing of relevant PCR amplicons. It is also possible that genetic variations from parental plants can alter PAMs and increase the potential off‐target effect in more complex and larger polyploid genomes like cotton (Li *et al*., 2018). Nevertheless, the results indicate that, with careful design of guide RNA and better knowledge of the genome, the CRISPR/Cas9 system can specifically target the pre‐selected sites for mutations.

### Grain numbers increase in *TaCKX2‐1* mutants

**Figure 7:**
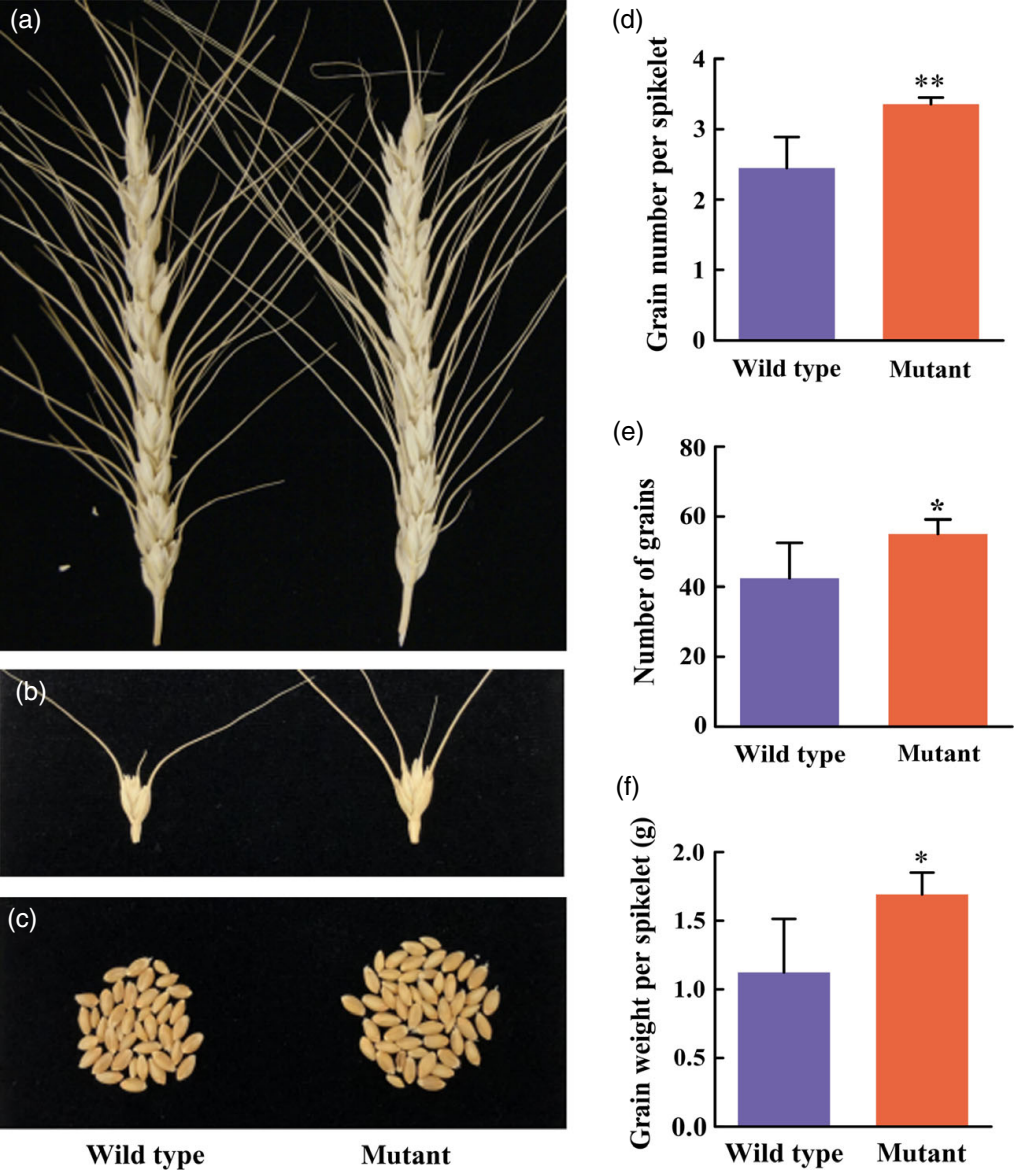
Effect the 1160‐bp deletion in *TaCKX2‐D1* on grain number. (a) Spikes of wild type (left) and the homozygous mutant (right) segregated from the T_2_ family #4‐6. (b) Spikelets of the wild type (left) and the homozygous mutant (right). (c) Seeds harvested from the main‐stem spikes of the wild type (left) and the homozygous mutant (right). (d) Grain number per spikelet of the wild type (left) and the homozygous mutant (right). The numbers in the *y*‐axis indicate grain number per spikelet. (e) Grain number per spike of the wild type (left) and the homozygous mutant (right). The numbers in the *y*‐axis indicate grain number per spike. (f) Grain weight per spike of the wild type (left) and the homozygous mutant (right). The numbers in the *y*‐axis indicate grain weight (gram) per spike. The error bars indicate the standard deviation of the mean calculated from five biological replicates.

While combining the mutations in the homoeologous copies of an orthologous gene or different orthologous loci, we evaluated the effect of mutation of the 1160‐bp deletion in *TaCKX2‐D1* on grain number per spikelet and spikelet density per spike. Results showed that there was no significant difference in spike length, spikelet number/spike, and spikelet density between the mutant and the wild type segregants from the same T_2_ population (*P *>* *0.76805). Compared to the wild type, the mutation increased grain number/spike from 42 to 55 (*P *=* *0.03374; Figure 7c,e), grain number/spikelet from 2.45 to 3.25 (*P *=* *0.00198; Figures 7b and 9d) and grain weight per spike from 1.12 to 1.69 g (*P *=* *0.01717; Figure 7f). These results indicate that *TaCKX2‐1* negatively regulates the trait of grain number in wheat.

## Discussion

### *Agrobacterium* delivery of CRISPR/Cas9 system

CRISPR/Cas9 has been the primary choice for plant genome editing, and *Agrobacterium*‐mediated transformation is the most common method for delivery of the CRISPR/Cas9 DNA components to plants (Soyars *et al*., 2018). Mainly due to historical reason, all genome editing systems in wheat were implemented through the biolistics‐mediated transformation (Liang *et al*., 2017; Sánchez‐León *et al*., 2018; Wang *et al*., 2014, 2018; Zhang *et al*., 2018a,2018b). Co‐transfer of large DNA fragments from the vector backbone by biolistic transformation leads to the high frequency of transgene silencing (Anand *et al*., 2003; Tassy *et al*., 2014). A large number of transgenic plants are required for recovery of desired edit mutations in T_0_ generation. Thus, transformation poses a bottleneck for many wheat genome editing projects and application in wheat breeding. By contrast, *Agrobacterium* mediation usually generates simple insertion events of the transfer DNA (T‐DNA), a DNA fragment defined by the border (left and right) sequences in a binary plasmid, with reduced frequency of transgene silencing (Dai *et al*., 2001). Recent progress in improving *Agrobacterium*‐mediated transformation efficiency (Ishida *et al*., 2015; Richardson *et al*., 2014) and expanding the spectrum of transformable genotypes (Wang *et al*., 2017a) makes it a more promising delivery method for wheat genome editing. In the present research, we developed an *Agrobacterium*‐delivered CRISPR/Cas9 system and successfully generated targeted mutations in four grain‐regulatory genes. The transgenes remain active and continuously induce site‐specific mutations in T_1_ and T_2_ generations. Compared to a limited number of T_0_ transgenic plants, it is much easier to generate large pools of T_1_ and T_2_ plants and screen for independent and novel mutations using the efficient and cheap Cas9/gRNA RNP assay. Thus, the *Agrobacterium*‐mediated system provides an alternative option for wheat genome editing, which requires a small number of transformation events but additional generations.

### Types and spectrum of edit mutations in wheat

Deep sequencing of the target region from the T_0_ plants and screening a large population of T_1_ and T_2_ plants give us the opportunity to investigate the spectrum and types of the edit mutations in wheat. Our results show that deletions are the dominant type of edit mutations in wheat. In T_0_ plants, deep sequencing indicates that over 99% of the mutations are due to deletions, and our genotyping data from T_1_ and T_2_ plants show that only five insertions were found of the 68 mutations detected, all of which are insertions of 1‐bp A or T. Remaining 63 mutations are deletions (Figure S11). Similar scenarios were also found in other wheat studies (Liang *et al*., 2017; Sánchez‐León *et al*., 2018; Wang *et al*., 2014, 2018; Zhang *et al*., 2018a,2018b). Thus, deletions are the dominant type of edit mutations in wheat. This scenario greatly contrasts with what was found in the model grass rice, where insertion is the primary type of edit mutations (Zhang *et al*., 2014). In the 68 mutants identified, deletions over 10 bp accounts for 73.5% and complex edit mutations account for 25% (Table S4; Figure S11). This contrasts the deep sequencing results from the T0 plants, where the 1‐, 2‐, and 37‐bp deletions are most abundant. This discrepancy might be associated with cell types wherein mutations occurred. The DNA used for deep sequencing was from leaf somatic cells, and the mutations recovered in the T_1_ and T_2_ plants occurred in germ cells or embryonic cells. Out of 180 T_2_ plants from the #4‐6 line, we detected 51 new mutations by PCR‐RE with an estimated edit rate of 28.3%. The estimated edit rate is much lower in *TaCKX2‐D1* as compared to *TaCKX‐A1* and *TaCKX‐B1* because the T_0_ plant #4 carried a 1160‐bp deletion allele and the T_1_ plant #4‐6 is heterozygous for this deletion. Therefore, the potential edit rate in *TaCKX2‐1* could be even higher. This is approximately threefold higher than the previously reports in wheat by Biolistic delivery (Liang *et al*., 2017; Sánchez‐León *et al*., 2018; Wang *et al*., 2014, 2018). Compared to *TaCKX2‐1* and *TaGW2*, the mutation rate in *TaGLW7* and *TaGW8* is much lower. This is because we intended to target the microRNA recognition sites of these two genes, which are highly conserved among the squarosa promoter binding‐like transcription factors (Xie *et al*., 2006). Thus, there is no other option for designing gene‐specific guide RNA genes. Despite the low edit efficiency compared to the model plants, we detected two homozygous mutants in *TaCKX2‐A1* and two triple mutants in a population of 231 T_2_ plants (Table S4 and S6).

### Use of edit mutations in wheat breeding

In addition to studies of gene function, CRISPR technology can have a great impact on plant breeding by manipulating the agriculturally important traits such as grain quality and grain yield. Our result showed that 1160‐bp deletion in *TaCKX2‐D1* significantly increased the grain number per spikelet. This is consistent with a prior study in rice, where reduction‐of‐function or loss‐of‐function mutations in *OsCKX2* (*Gn1*) increased grain number per panicle (Ashikari *et al*., 2005). The effect of *TaCKX2‐1* on grain number needs to be further validated by using other independent homozygous mutants of single double or triple mutations of the same orthologous gene. In addition to *TaCKX2‐1*, we also obtained mutation in three other grain regulatory genes, *TaGLW7*,* TaGW2*, and *TaGW8*. Two recent studies on *TaGW2* showed that knockout mutations of this gene could significantly increase wheat grain size and grain weight (Wang *et al*., 2018; Zhang *et al*., 2018a,2018b). Combining and transferring the mutations of *TaCKX2‐1* and *TaGW2* into the elite wheat cultivars are expected to have an additive effect on increasing the sink strength.

Wheat cultivars Bobwhite and Fielder are the most transformable genotypes, but they are outdated and not currently used in wheat production and by wheat breeding. For the use of the edit mutations in wheat improvement, two approaches may be considered. A straightforward approach would be transferring the desired mutation alleles into elite genotypes and purging the CRISPR/Cas9 transgene by backcrossing. Another approach would be transferring the CRISPR/Cas9 transgene into the elite cultivars and screen new mutations in the improved background. Compared to the former, the latter approach would reduce the risk of genetic vulnerability due to the reduction in diversity in the region surrounding the mutant alleles. One prerequisite for this approach is the inheritance of active CRISPR/Cas9 transgene through the backcrossing generations. In this respect, the *Agrobacterium*‐mediated delivery system has an advantage over the biolistic delivery as demonstrated by the present research that the CRISPR/Cas9 transgene remains high activity in the T_1_ and T_2_ generation. Both approaches can be coupled with genomic selection for acceleration of the transgene‐free mutants as novel germplasm or cultivars.

## Experimental procedures

### Plants and growing conditions

Wheat cultivar Fielder and its transgenic plants were grown in 6″ pots containing Sunshine potting mix (Green State Gardener, Burlington, VT) mixed with Perlite and Vermiculite (Hummert, St Louis, MO) in equal ratio and supplied with Multicote controlled release fertilizer (Haifa Group, Haifa, Israel), one plant per pot. Plants were grown in greenhouse in the fall, winter and spring seasons and in growth chambers in summer to increase tillers. The temperature of the greenhouse and growth chambers was set at 22 °C with a 16 h of photoperiod, and the plants were watered every other day.

### Construction of plasmids

Wheat U6 snRNA (Marshallsay *et al*., 1992) was used as a query to BLAST search against wheat reference genome IWGSC (v1.0) (International Wheat Genome Sequencing Consortium, 2018) to retrieve their promoter sequences. gBclocks for promoters of TaU6.1 and TaU6.2 were synthesized (Integrated DNA Technology, Iowa City, IA) while TaU6.3 and TaU6.5 were PCR amplified from wheat genomic DNA. U6 promoters were then cloned into pENTR4 together with guide RNA (gRNA) scaffold and U6 terminator, which is flanked by *att*L1 and *att*L2 sites. Two *Btg*ZI restriction sites or two *Bsa*I restriction sites were introduced during the gBlock synthesis for cloning of the sgRNA spacer sequences. After testing the efficiency of U6 promoters (Appendix S1) in wheat protoplasts, two most active U6 promoters, i.e., U6.1 and U6.3 promoters, were assembled into the same construct to facilitate the construction of two sgRNA genes in an intermediate vector, pTagRNA4.

Two versions of Cas9 were used. The first Cas9 was codon‐optimized for expression in wheat and made from six gBlocks through the Gibson assembly using Gibson Assembly^®^ Master Mix (New England Biolabs, Ipswich, MA). The assembled Cas9 was cloned into pMCG1005 in which the selection marker *Bar* gene was replaced with *hygromycin phosphotransferase* (*hpt*) gene. After confirmation of the *hpt* activity in rice callus through *Agrobacterium*‐mediated transformation, the construct was inserted with the *Att*R1‐*ccdB*‐*Att*R2 Gateway cassette to produce plasmid pTaCas9‐Hyg‐GW. The selection efficiency of the *ccdB* gene was examined by the introduction of the plasmid into *Escherichia coli* XL1‐blue (Agilent Genomics, Santa Clara, CA) for minimal colony formation. pTaCas9‐Hyg‐GW was used for testing the activity of U6 promoters in protoplasts. The second Cas9 gene, Cas9:P2A:H2A:GFP and referred to as Cas9‐GFP hereinafter, was also wheat‐codon optimized and derived from the fusion of Cas9 with the histone 2A (H2A) and GFP coding sequences, the Cas9 coding sequence was separated by a sequence encoding P2A, a translational slipping signal. This Cas9‐GFP under the maize *ubiquitin 1* gene promoter was used for wheat transgenics.

To transiently and quickly identify active U6 promoters for gRNA, a mutated *green fluorescent protein* (*GFP*) gene was constructed. To insert a 1‐bp downstream of the start codon of *GFP*, a forward primer GFP+1‐F, with the frameshift insertion, was paired with the reverse primer GFP‐R (Table S9) to amplify the open reading frame (ORF) of the *GFP* gene. The PCR product was digested with *Bam*HI and *Spe*I and cloned into pUC19 under the 35S promoter, resulting in pUC‐35S:GFP+1. A sgRNA targeting the 1‐bp insertion site in pUC‐35S:GFP+1 was synthesized and cloned into the gRNA vector to produce the construct pTagRNA4‐gGFP.

To make the constructs targeting the grain regulatory genes in wheat, two complementary oligonucleotides corresponding to the protospacer sequences of each of two sgRNAs, sgRNA1 and sgRNA2 for the same gene, were designed and synthesized. Double‐stranded oligonucleotides were sequentially cloned into *Btg*ZI sites and *Bsa*I sites in pTagRNA4, in which sgRNA1 is driven by TaU6.1 and sgRNA2 by TaU6.3. gRNA cassette was then PCR‐amplified and integrated into pCas9‐GFP (Cas9‐GFP in pENTR4) at the *Bss*HII site by Gibson assembly. Through Gateway recombination reaction, the Cas9‐TagRNA4 cassette was finally incorporated into pLC41‐GW (a Gateway binary vector) that is derived from pLC41_Exp1 Icat‐GUS.

To make an *in vitro* transcription system for guide RNA that can be used along with Cas9 protein to *in vitro* screen for mutated plants, a gBlock containing the T7 promoter, guide RNA scaffold, and two *Bsa*I cloning sites in between was synthesized (Integrated DNA Technology, Iowa City, IA). The gBlock was inserted into pENTR4 between *Nco*I and *Xba*I through Gibson assembly replacing the *ccdB* cassette, resulting in pT7‐sgRNA. To make *in vitro* transcribed guide RNA molecule, a guide RNA gene was assembled in pT7‐sgRNA vector. Specifically, two complementary oligonucleotides for the spacer sequence of sgRNAs were synthesized, annealed to form double stranded DNA fragment, and then cloned into *Bsa*I sites in pT7‐sgRNA.

### Isolation and transfection of wheat protoplasts

Leaves from 1‐week‐old seedlings of wheat cultivar Fielder were chopped latitudinally into 0.5‐mm strips using razor blades. The leaf strips were used for protoplast isolation following the procedure described by Zhou *et al*. (2014). The protoplasts were resuspended in a W5 solution (2 mm MES (pH 5.7), 154 mm NaCl, 125 mm CaCl_2_, 5 mm KCl) at a concentration of 2 × 10^6^ per mL. The pTaCas9‐Hyg‐GW, pUC‐35S:GFP+1, and pTaU6‐gGFP were mixed at a concentration of 400 ng/μL each plasmid. An aliquot of 200 μL protoplast solution was sequentially mixed with 30 μL of plasmid DNA and 230 μL of freshly prepared PEG solution (40% PEG‐4000, 0.2 m Mannitol, 0.1 m CaCl_2_) in a 2‐ml microcentrifuge tube by inverting three times. After the mixture was incubated at room temperature in the dark for 20 min, 900 μL of W5 solution was added to the tube and mixed by inverting the tube three times. The transfected protoplasts were collected by centrifugation at 250 g for 5 min, washed once with W5 solution, re‐suspended in 800 μL of W5 solution, and incubated at room temperature. Constructs pOsUbi‐GFP and pUC‐35S:GFP+1 served as a positive and negative control, respectively, to transfect the protoplasts. Forty hours after transfection, protoplasts were examined under a fluorescence microscope for the proportion of protoplasts with GFP fluorescence and images were captured. Transfection was conducted with two replicates for each construct, and five random microscope views under 20× magnification were calculated. Images were analysed with Fiji‐ImageJ (Schindelin *et al*., 2012), and integrated density was measured to quantify the fluorescence density in the selected view. Integrated density was then averaged by the total number of protoplasts in that view.

### Primers and guide RNA design

Total genomic DNA was isolated from the wheat leaf tissue following the protocol described by Li *et al*. (2008) except using the CTAB buffer (2% CTAB (hexadecyl trimethyl‐ammonium bromide), 100 mm Tris, 20 mm EDTA, 1.4 m NaCl, 1% PVP (polyvinyl pyrrolidone (vinylpyrrolidine homopolymer) Mw 40 000), and pH5.0). Sequences of the homoeologous genes of *T. aestivum* cv Chinese Spring (CS) were retrieved from the Ensembl Plants database (https://plants.ensembl.org/index.html), and gene‐specific primers (Table S9) were designed for the homoeologous genes using online program GSP (https://probes.pw.usda.gov/GSP/; Wang *et al*., 2016) and used to PCR‐amplify the relevant genomic regions (~1 kb). Specificity of the primers was validated by use of DNA from the nulli‐tetrasomic stocks (Sears, 1966), and the PCR products were separated through electrophoresis in 1% agarose gel. All the primers are listed in Table S9. The PCR reaction was performed for each homoeologous copy of an orthologous gene in the A, B, and D genome, which included 100 ng genomic DNA, 5× GoTaq^®^ Green PCR buffer (Promega, Madison, WI), 125 μm dNTPs, 0.5 μm primers, and 0.2 μL DNA Taq polymerase. PCR cycling conditions were as follows: an initial denaturation at 95 °C for 5 min, followed by 35 cycles of 95 °C for 30 s, 55 °C for 30 s, and 72 °C for 1.5 min, and a final extension at 72 °C for 7 min. The gene‐specific PCR products were cleaned up with ExoSAP‐IT (Thermo Fisher Scientific, Waltham, MA) treatment and subjected to sequencing. The sequences from the A, B, and D genome were aligned using the online program Multiple Sequence Alignment (MUSCLE, https://www.ebi.ac.uk/Tools/msa/muscle/) and conserved sequences in the first exon of the genes were further inspected for selection of 20 nucleotides immediately upstream of a PAM motif (5′‐NGG‐3′) and a GC content of 40%–60% for guide RNA design. All guide RNA target sequences are listed in Table S7.

### Wheat transformation

Fielder was cultivated in a growth chamber. Immature seeds were harvested from panicles approximately 2 weeks after anthesis, surface‐sterilized for 1 min in 70% ethanol followed by 10 min in 1.2% sodium hypochlorite and were washed three times with sterilized water. Immature embryos were isolated from the sterilized seeds under a stereoscopic microscope. The CRISPR/Cas9 constructs were transferred into *Agrobacterium tumefaciens* strain EHA105 by electroporation. The *Agrobacterium*‐mediated transformation on the isolated immature embryos and plant regeneration were conducted following the protocol described by Ishida *et al*. (2015). The transgenic plants were further confirmed by PCR assays of the Cas9 and sgRNA transgenes.

### Purification of Cas9 protein, *in vitro* transcription of guide RNA and preparation of Cas9/sgRNA ribonucleoprotein

*Escherichia coli* strain Rosetta 2 DE3 carrying plasmid pMJ806 was obtained from Addgene. The Cas9 protein was prepared following the protocol as described (Anders and Jinek, 2014). For *in vitro* transcription of guide RNA, pT7‐sgRNA derived gRNA‐specific construct was linearized at a unique restriction enzyme site *Nco*I, followed by dephosphorylation with Shrimp Alkaline Phosphatase (rSAP) (New England Biolabs, Ipswich, MA), and then purified with DNA Clean and Concentrator Kit (Zymo Research, Irvine, CA). sgRNA was synthesized by using the HiScribe T7 Quick High Yield RNA Synthesis Kit (New England Biolabs) following the manufacturer's instructions and purified with RNA Clean and Concentrator (Zymo Research, Irvine, CA). For each DNA/ribonucleoprotein reaction, 1 μg of sgRNA and 1 μg of Cas9 protein were mixed in Cas9 reaction buffer and incubated at room temperature for 15 min to form a ribonucleoprotein (RNP) complex. The resulting RNP was used for 2–3 reactions each with about 1 μg of PCR product in a total volume of 10 μL at 37 °C for 3 h. After inactivated at 65 °C for 15 min, the reactions were analysed with electrophoresis in 2% agarose gel.

### Mutant screening

The PCR amplicons were digested with restriction enzymes (*Xcm*I and *Bgl*I), T7E1 or Cas9/sgRNA ribonucleoprotein and separated by agarose electrophoresis. For digestion with restriction enzymes or T7E1, 1 unit of enzyme was used in 15 μL reaction including PCR product, following the manufacturer's instruction. After being denatured (95 °C for 5 min) and re‐annealed (ramp down to 25 °C at 5 °C/min), the PCR products were subjected to T7E1 (New England Biolabs, Ipswich, MA) digestion (1 unit, 0.2 μL) at 37°C for 1.5 h and separated by agarose gel electrophoresis.

### DNA sequencing and sequence analyses

The PCR products were deep‐sequenced by the high‐throughput sequencing platform HiSeq 2000 (Illumina, San Diego, CA) or individually sequenced by Sanger sequencing in the ABI DNA analysers (Thermo Fisher Scientific, Waltham, MA). Genomic DNA samples from T_0_ plants transformed with *Cas9* and *TaCKX2‐1* sgRNA was used in amplicon sequencing. First round PCR was conducted using gene‐specific primers with universal tags for 35 cycles. PCR products were separated in 2.0% agarose gel, and the target bands were excised and column‐purified. Approximately 10 ng of PCR product was used for a second round of PCR with primers matching the universal tags and containing a sample‐specific barcode within the forward primer. PCR was conducted for 12 cycles. PCR products were separated on 2.0% agarose gel, and target bands were cut and column‐purified. Purified amplicons were quantified with Qubit^®^ 2.0 fluorometer (Life Technologies, Carlsbad, CA) and pooled equally to make a library at a concentration of 4 nmol/L. Paired‐end (2 × 250) sequencing was conducted with a total of 500 cycles. Sequence analysis was conducted with CRISPR‐DAV (Wang *et al*., 2017a, 2017b) to discover the modification.

For Sanger sequencing, PCR amplicons were treated with ExoSAP‐IT™ PCR Product Cleanup Reagent (Thermo Fisher) following the manufacturer's manual before subjected to sequencing. The sequencing chromatograms were fed to the computer program DSDecodeM (http://skl.scau.edu.cn/dsdecode/; Liu *et al*., 2015) to determine the allelic mutations.

### Phenotyping

The spikes from the main stems were scored for spikelet and grain numbers; spikelet length, density and average grain number per spikelet were measured. The total grains from individual spike were weighed using a PB602‐S digital precision scale (Mettler Toledo, Columbus, OH). Five biological replicates were used for each genotype.

### Data analysis

The Student's *t*‐test was performed using pooled standard deviations to evaluate the statistical significance of the difference between different U6 promoters or between the mutant and wild type. Chisq test was implemented for fitness of segregation of mutations in a T_2_ population to the 1 : 2 : 1 ratio. Both the Student's and Chisq tests were conducted using Microsoft Excel. The cut‐off for statistical significance was set to a *P*‐value of 0.05.

## Conflict of interest

The authors declare no conflicts of interest.

## Supporting information


**Figure S1** Comparison in editing efficiency of gGFP driven by different wheat U6 promoters in terms of the proportion of fluorescence cell (a) and average fluorescence intensity per cell (b).
**Figure S2** Structure of pTagRNA4 in the pENTR4 backbone. Two sgRNAs are inserted into *Btg*ZI‐*Btg*ZI and *Bsa*I‐*Bsa*I sites sequentially.
**Figure S3** Structure of pCas9‐GFP used in the transgene. pTaU6‐gRNA cassette is inserted into *Bss*HII sites through Gibson assembly.
**Figure S4** Deletion distribution along the amplicon in *TaCKX2‐A1* (a), *TaCKX2‐B1* (b), and *TaCKX2‐D1* (c).
**Figure S5** Identification of *Cas9*‐positive transgenic plants in T_1_ progenies.
**Figure S6** Targeted *TaCKX2‐1* homoeologues using the CRISPR/Cas9 system in transgenic T_1_ plants.
**Figure S7** Screen mutant plants in *TaCKX2‐D1* # 4 T_1_ plants using PCR assay.
**Figure S8** Screen mutations in *TaCKX2‐1* of plants from the # 4‐1 and # 4‐35 T_2_ family by the PCR‐RE assay.
**Figure S9** Screen mutations in *TaCKX2‐1* of the T_2_ plants from the # 4‐6 family by the PCR‐RE assay.
**Figure S10** Screening of mutations in *TaGLW7* of the T_2_ plants from the # 1‐32 family by the PCR‐RNP assay.
**Figure S11** Types and spectrum of 68 edit mutations.

**Table S1** sgRNA target sites used in this study.
**Table S2** Verification of transgenes in T_0_ plants.
**Table S3** Frequencies of CRISPR/Cas9‐induced novel mutations in T_0_, T_1_, and T_2_ populations.
**Table S4** Types of CRISPR/Cas9‐induced mutations for target genes in T_0_, T_1_, and T_2_ generations.
**Table S5** Genotypes of mutant plants induced by CRISPR/Cas9 system in T_1_ population.
**Table S6** Genotypes of mutant plants induced by CRISPR/Cas9 system in *TaCKX2* T2 family #4‐6.
**Table S7** A summary of the double and triple mutants recovered in *TaCKX2‐1*.
**Table S8** Off‐targeting of designed sgRNA for *TaCKX2‐1* gene.
**Table S9** PCR primers used in this study.

**Appendix S1** Sequences of U6 promoters.

## References

[pbi13088-bib-0001] Anand, A. , Zhou, T. , Trick, H.N. , Gill, B.S. , Bockus, W.W. and Muthukrishnan, S. (2003) Greenhouse and field testing of transgenic wheat plants stably expressing genes for thaumatin‐like protein, chitinase and glucanase against *Fusarium graminearum* . J. Exp. Bot. 54, 1101–1111. 12598580 10.1093/jxb/erg110

[pbi13088-bib-0002] Anders, C. and Jinek, M. (2014) In vitro enzymology of Cas9. Methods Enzymol. 546, 1–20. 25398333 10.1016/B978-0-12-801185-0.00001-5 PMC5074358

[pbi13088-bib-0003] Ashikari, M. , Sakakibara, H. , Lin, S. , Yamamoto, T. , Takashi, T. , Nishimura, A. , Angeles, E.R. *et al* (2005) Cytokinin oxidase regulates rice grain production. Science, 309, 741–745. 15976269 10.1126/science.1113373

[pbi13088-bib-0004] Cai, C.Q. , Doyon, Y. , Ainley, W.M. , Miller, J.C. , Dekelver, R.C. , Moehle, E.A. , Rock, J.M. *et al* (2009) Targeted transgene integration in plant cells using designed zinc finger nucleases. Plant Mol. Biol. 69, 699–709. 19112554 10.1007/s11103-008-9449-7

[pbi13088-bib-0005] Carroll, D. (2011) Genome engineering with zinc‐finger nucleases. Genetics, 188, 773–782. 21828278 10.1534/genetics.111.131433 PMC3176093

[pbi13088-bib-0006] Cermak, T. , Doyle, E.L. , Christian, M. , Wang, L. , Zhang, Y. , Schmidt, C. , Baller, J.A. *et al* (2011) Efficient design and assembly of custom TALEN and other TAL effector‐based constructs for DNA targeting. Nucleic Acids Res. 39, e28. 21493687 10.1093/nar/gkr218 PMC3130291

[pbi13088-bib-0007] Christian, M. , Qi, Y. , Zhang, Y. and Voytas, D.F. (2013) Targeted mutagenesis of *Arabidopsis thaliana* using engineered TAL effector nucleases. G3 (Bethesda), 3, 1697–16705. 23979944 10.1534/g3.113.007104 PMC3789794

[pbi13088-bib-0008] Clasen, B.M. , Stoddard, T.J. , Luo, S. , Demorest, Z.L. , Li, J. , Cedrone, F. , Tibebu, R. *et al* (2015) Improving cold storage and processing traits in potato through targeted gene knockout. Plant Biotechnol. J. 14, 169–176. 25846201 10.1111/pbi.12370 PMC11389148

[pbi13088-bib-0009] Cong, L. , Ran, F.A. , Cox, D. , Lin, S. , Barretto, R. , Habib, N. , Hsu, P.D. *et al* (2013) Multiplex genome engineering using CRISPR/Cas systems. Science, 339, 819–823. 23287718 10.1126/science.1231143 PMC3795411

[pbi13088-bib-0010] Dai, S. , Zheng, P. , Marmey, P. , Zhang, S. , Tian, W. , Chen, S. , Beachy, R.N. *et al* (2001) Comparative analysis of transgenic rice plants obtained by *Agrobacterium*‐mediated transformation and particle bombardment. Mol. Breed. 7, 25–33.

[pbi13088-bib-0011] Gaudelli, N.M. , Komor, A.C. , Rees, H.A. , Packer, M.S. , Badran, A.H. , Bryson, D.I. and Liu, D.R. (2017) Programmable base editing of A•T to G•C in genomic DNA without DNA cleavage. Nature, 551, 464–471. 29160308 10.1038/nature24644 PMC5726555

[pbi13088-bib-0012] Haun, W. , Coffman, A. , Clasen, B.M. , Demorest, Z.L. , Lowy, A. , Ray, E. , Retterath, A. *et al* (2014) Improved soybean oil quality by targeted mutagenesis of the fatty acid desaturase 2 gene family. Plant Biotechnol. J. 12, 934–940. 24851712 10.1111/pbi.12201

[pbi13088-bib-0013] International Wheat Genome Sequencing Consortium (2018) A chromosome‐based draft sequence of the hexaploid bread wheat (*Triticum aestivum*) genome. Science, 345, 1251788. 10.1126/science.1251788 25035500

[pbi13088-bib-0014] Ishida, Y. , Tsunashida, M. , Hiei, Y. and Komari, T. (2015) Wheat (*Triticum aestivum* L.) transformation using immature embryos. Methods Mol. Biol. 1223, 189–198. 25300841 10.1007/978-1-4939-1695-5_15

[pbi13088-bib-0015] Jinek, M. , Chylinski, K. , Fonfara, I. , Hauer, M. , Doudna, J.A. and Charpentier, E. (2012) A programmable dual‐RNA‐guided DNA endonuclease in adaptive bacterial immunity. Science, 337, 816–821. 22745249 10.1126/science.1225829 PMC6286148

[pbi13088-bib-0016] Jinek, M. , Jiang, F. , Taylor, D.W. , Sternberg, S.H. , Kaya, E. , Ma, E. , Anders, C. *et al* (2014) Structures of Cas9 endonucleases reveal RNA‐mediated conformational activation. Science, 343, 1247997. 24505130 10.1126/science.1247997 PMC4184034

[pbi13088-bib-0017] Komor, A.C. , Kim, Y.B. , Packer, M.S. , Zuris, J.A. and Liu, D.R. (2016) Programmable editing of a target base in genomic DNA without double‐stranded DNA cleavage. Nature, 533, 420–424. 27096365 10.1038/nature17946 PMC4873371

[pbi13088-bib-0018] Li, W. and Yang, B. (2017) Translational genomics of grain size regulation in wheat. Theor. Appl. Genet. 130, 1765–1771. 28765985 10.1007/s00122-017-2953-x

[pbi13088-bib-0019] Li, W. , Huang, L. and Gill, B.S. (2008) Recurrent deletions of puroindoline genes at the grain hardness locus in four independent lineages of polyploid wheat. Plant Physiol. 146, 200–212. 18024553 10.1104/pp.107.108852 PMC2230614

[pbi13088-bib-0020] Li, T. , Huang, S. , Jiang, W.Z. , Wright, D. , Spalding, M.H. , Weeks, D.P. and Yang, B. (2011) TAL nucleases (TALNs): hybrid proteins composed of TAL effectors and FokI DNA‐cleavage domain. Nucleic Acids Res. 39, 359–372. 20699274 10.1093/nar/gkq704 PMC3017587

[pbi13088-bib-0021] Li, T. , Liu, B. , Spalding, M.H. , Weeks, D.P. and Yang, B. (2012) High‐efficiency TALEN‐based gene editing produces disease‐resistant rice. Nat. Biotechnol. 30, 390–392. 22565958 10.1038/nbt.2199

[pbi13088-bib-0022] Li, J. , Manghwar, H. , Sun, L. , Wang, P. , Wang, G. , Sheng, H. , Zhang, J. *et al* (2018) Whole genome sequencing reveals rare off‐target mutations and considerable inherent genetic or/and somaclonal variations in CRISPR/Cas9‐edited cotton plants. Plant Biotechnol. J. 10.1111/pbi.13020. PMC6587709 30291759

[pbi13088-bib-0023] Liang, Z. , Chen, K. , Li, T. , Zhang, Y. , Wang, Y. , Zhao, Q. , Liu, J. *et al* (2017) Efficient DNA‐free genome editing of bread wheat using CRISPR/Cas9 ribonucleoprotein complexes. Nat. Commun. 8, 14261. 28098143 10.1038/ncomms14261 PMC5253684

[pbi13088-bib-0024] Liu, W. , Xie, X. , Ma, X. , Li, J. , Chen, J. and Liu, Y.G. (2015) DSDecode: a web‐based tool for decoding of sequencing chromatograms for genotyping of targeted mutations. Mol. Plant, 8, 1431–1433. 26032088 10.1016/j.molp.2015.05.009

[pbi13088-bib-0025] Maeder, M.L. , Thibodeau‐Beganny, S. , Osiak, A. , Wright, D.A. , Anthony, R.M. , Eichtinger, M. , Jiang, T. *et al* (2008) Rapid “open‐source” engineering of customized zinc‐finger nucleases for highly efficient gene modification. Mol. Cell, 31, 294–301. 18657511 10.1016/j.molcel.2008.06.016 PMC2535758

[pbi13088-bib-0026] Mahfouz, M.M. , Li, L. , Shamimuzzaman, M. , Wibowo, A. , Fang, X. and Zhu, J.‐K. (2011) De novo‐engineered transcription activator‐like effector (TALE) hybrid nuclease with novel DNA binding specificity creates double‐strand breaks. Proc. Natl Acad. Sci. USA, 108, 2623–2628. 21262818 10.1073/pnas.1019533108 PMC3038751

[pbi13088-bib-0027] Mali, P. , Yang, L. , Esvelt, K.M. , Aach, J. , Guell, M. , DiCarlo, J.E. , Norville, J.E. *et al* (2013) RNA‐guided human genome engineering via Cas9. Science, 339, 823–826. 23287722 10.1126/science.1232033 PMC3712628

[pbi13088-bib-0028] Marshallsay, C. , Connelly, S. and Filipowicz, W. (1992) Characterization of the U3 and U6 snRNA genes from wheat: U3 snRNA genes in monocot plants are transcribed by RNA polymerase III. Plant Mol. Biol. 19, 973–983. 1511142 10.1007/BF00040529

[pbi13088-bib-0029] Nishida, K. , Arazoe, T. , Yachie, N. , Banno, S. , Kakimoto, M. , Tabata, M. , Mochizuki, M. *et al* (2016) Targeted nucleotide editing using hybrid prokaryotic and vertebrate adaptive immune systems. Science, 353, aaf8729. 27492474 10.1126/science.aaf8729

[pbi13088-bib-0030] Richardson, T. , Thistleton, J. , Higgins, T.J. , Howitt, C. and Ayliffe, M. (2014) Efficient *Agrobacterium* transformation of elite wheat germplasm without selection. Plant Cell Tiss. Organ. Cult. 119, 647–659.

[pbi13088-bib-0031] Sánchez‐León, S. , Gil‐Humanes, J. , Ozuna, C.V. , Giménez, M.J. , Sousa, C. , Voytas, D.F. and Barro, F. (2018) Low‐gluten, nontransgenic wheat engineered with CRISPR/Cas9. Plant Biotechnol. J. 16, 902–910. 28921815 10.1111/pbi.12837 PMC5867031

[pbi13088-bib-0032] Schindelin, J. , Arganda‐Carreras, I. , Frise, E. , Kaynig, V. , Longair, M. , Pietzsch, T. , Preibisch, S. *et al* (2012) Fiji: an open‐source platform for biological‐image analysis. Nat. Methods, 9, 676–682. 22743772 10.1038/nmeth.2019 PMC3855844

[pbi13088-bib-0033] Sears, E.R. (1966) Nullisomic‐tetrasomic combinations in hexaplold wheat In Chromosome Manipulation and Plant Genetics (Lewis D.R., ed.), pp. 29–47. London: Oliver and Boyd.

[pbi13088-bib-0034] Shan, Q. , Wang, Y. , Chen, K. , Liang, Z. , Li, J. , Zhang, Y. , Zhang, K. *et al* (2013a) Rapid and efficient gene modification in rice and *Brachypodium* using TALENs. Mol. Plant, 6, 365–368. 10.1093/mp/sss162 PMC3968307 23288864

[pbi13088-bib-0035] Shan, Q. , Wang, Y. , Li, J. , Zhang, Y. , Chen, K. , Liang, Z. , Zhang, K. *et al* (2013b) Targeted genome modification of crop plants using a CRISPR‐Cas system. Nat. Biotechnol. 31, 686–688. 23929338 10.1038/nbt.2650

[pbi13088-bib-0036] Soyars, C.L. , Peterson, B.A. , Burr, C.A. and Nimchuk, Z.L. (2018) Cutting edge genetics: CRISPR/Cas9 editing of plant genomes. Plant Cell Physiol. 59, 1608–1620. 29912402 10.1093/pcp/pcy079

[pbi13088-bib-0037] Symington, L.S. and Gautier, J. (2011) Double‐strand break end resection and repair pathway choice. Annu. Rev. Genet. 45, 247–271. 21910633 10.1146/annurev-genet-110410-132435

[pbi13088-bib-0038] Tassy, C. , Partier, A. , Beckert, M. , Feuillet, C. and Barret, P. (2014) Biolistic transformation of wheat: increased production of plants with simple insertions and heritable transgene expression. Plant Cell, Tissue Organ Cult. 119, 171–181.

[pbi13088-bib-0039] Wang, Y. , Cheng, X. , Shan, Q. , Zhang, Y. , Liu, J. , Gao, C. and Qiu, J. (2014) Simultaneous editing of three homoeoalleles in hexaploid bread wheat confers heritable resistance to powdery mildew. Nat. Biotechnol. 32, 947–951. 25038773 10.1038/nbt.2969

[pbi13088-bib-0501] Wang, Y. , Tiwari, V.K. , Rawat, N. , Gill, B.S. , Huo, N. , You, F.M. , Coleman‐Derr, D. , *et al* (2016) GSP: a web‐based platform for designing genome‐specific primers in polyploids. Bioinformatics 32, 2382‐2383. 27153733 10.1093/bioinformatics/btw134

[pbi13088-bib-0040] Wang, K. , Liu, H. , Du, L. and Ye, X. (2017a) Generation of marker‐free transgenic hexaploid wheat via an *Agrobacterium*‐mediated co‐transformation strategy in commercial Chinese wheat varieties. Plant Biotechnol. J. 15, 614–623. 27862820 10.1111/pbi.12660 PMC5399001

[pbi13088-bib-0041] Wang, X. , Tilford, C. , Neuhaus, I. , Mintier, G. , Guo, Q. , Feder, J.N. and Kirov, S. (2017b) CRISPR‐DAV: CRISPR NGS data analysis and visualization pipeline. Bioinformatics, 33, 3811–3812. 28961906 10.1093/bioinformatics/btx518

[pbi13088-bib-0042] Wang, W. , Pan, Q. , He, F. , Akhunova, A. , Chao, S. , Trick, H.A. and Akhunov, E. (2018) Transgenerational CRISPR‐Cas9 activity facilitates multiplex gene editing in allopolyploid wheat. CRISPR J. 1, 65–74. 30627700 10.1089/crispr.2017.0010 PMC6319321

[pbi13088-bib-0043] Weeks, D.P. , Spalding, M.H. and Yang, B. (2016) Use of designer nucleases for targeted gene and genome editing in plants. Plant Biotechnol. J. 14, 483–495. 26261084 10.1111/pbi.12448 PMC11388832

[pbi13088-bib-0044] Wright, D.A. , Townsend, J.A. , Winfrey, R.J.J. , Irwin, P.A. , Rajagopal, J. , Lonosky, P.M. , Hall, B.D. *et al* (2005) High‐frequency homologous recombination in plants mediated by zinc‐finger nucleases. Plant J. 44, 693–705. 16262717 10.1111/j.1365-313X.2005.02551.x

[pbi13088-bib-0045] Xie, K. , Wu, C. and Xiong, L. (2006) Genomic organization, differential expression, and interaction of SQUAMOSA promoter‐binding‐like transcription factors and microRNA156 in rice. Plant Physiol. 142, 280–293. 16861571 10.1104/pp.106.084475 PMC1557610

[pbi13088-bib-0046] Zhang, H. , Zhang, J. , Wei, P. , Zhang, B. , Gou, F. , Feng, Z. , Mao, Y. *et al* (2014) The CRISPR/Cas9 system produces specific and homozygous targeted gene editing in rice in one generation. Plant Biotechnol. J. 12, 797–807. 24854982 10.1111/pbi.12200

[pbi13088-bib-0047] Zhang, H. , Gou, F. , Zhang, J. , Liu, W. , Li, Q. , Mao, Y. , Botella, J.R. *et al* (2015) TALEN‐mediated targeted mutagenesis produces a large variety of heritable mutations in rice. Plant Biotechnol. J. 14, 186–194. 25867543 10.1111/pbi.12372 PMC11388857

[pbi13088-bib-0048] Zhang, S. , Zhang, R. , Song, G. , Gao, J. , Li, W. , Han, X. , Chen, M. *et al* (2018a) Targeted mutagenesis using the *Agrobacterium tumefaciens*‐mediated CRISPR‐Cas9 system in common wheat. BMC Plant Biol. 18, 302. 30477421 10.1186/s12870-018-1496-x PMC6258442

[pbi13088-bib-0049] Zhang, Y. , Li, D. , Zhang, D. , Zhao, X. , Cao, X. , Dong, L. , Liu, J. *et al* (2018b) Analysis of the functions of TaGW2 homoeologs in wheat grain weight and protein content traits. Plant J. 94, 857–866. 29570880 10.1111/tpj.13903

[pbi13088-bib-0050] Zhou, H. , Liu, B. , Weeks, D.P. , Spalding, M.H. and Yang, B. (2014) Large chromosomal deletions and heritable small genetic changes induced by CRISPR/Cas9 in rice. Nucleic Acids Res. 42, 10903–10914. 25200087 10.1093/nar/gku806 PMC4176183

