# Supplementary material for: Development of an Agrobacterium‐delivered CRISPR/Cas9 system for wheat genome editing
Source: Plant Biotechnol J. 2019 Mar 12;17(8):1623–35. doi: 10.1111/pbi.13088 (PMC6662106; doi:10.1111/pbi.13088)
Supplement: Supplementary file 1 — Figure S1 Comparison in editing efficiency of gGFP driven by different wheat U6 promoters in terms of the proportion of fluorescence cell (a) and average fluorescence intensity per cell (b). Figure S2 Structure of pTagRNA4 in the pENTR4 backbone. Two sgRNAs are inserted into BtgZI‐BtgZI and BsaI‐BsaI sites sequentially. Figure S3 Structure of pCas9‐GFP used in the transgene. pTaU6‐gRNA cassette is inserted into BssHII sites through Gibson assembly. Figure S4 Deletion distribution along the amplicon in TaCKX2‐A1 (a), TaCKX2‐B1 (b), and TaCKX2‐D1 (c). Figure S5 Identification of Cas9‐positive transgenic plants in T1 progenies. Figure S6 Targeted TaCKX2‐1 homoeologues using the CRISPR/Cas9 system in transgenic T1 plants. Figure S7 Screen mutant plants in TaCKX2‐D1 # 4 T1 plants using PCR assay. Figure S8 Screen mutations in TaCKX2‐1 of plants from the # 4‐1 and # 4‐35 T2 family by the PCR‐RE assay. Figure S9 Screen mutations in TaCKX2‐1 of the T2 plants from the # 4‐6 family by the PCR‐RE assay. Figure S10 Screening of mutations in TaGLW7 of the T2 plants from the # 1‐32 family by the PCR‐RNP assay. Figure S11 Types and spectrum of 68 edit mutations. [file PBI-17-1623-s001.docx]

**
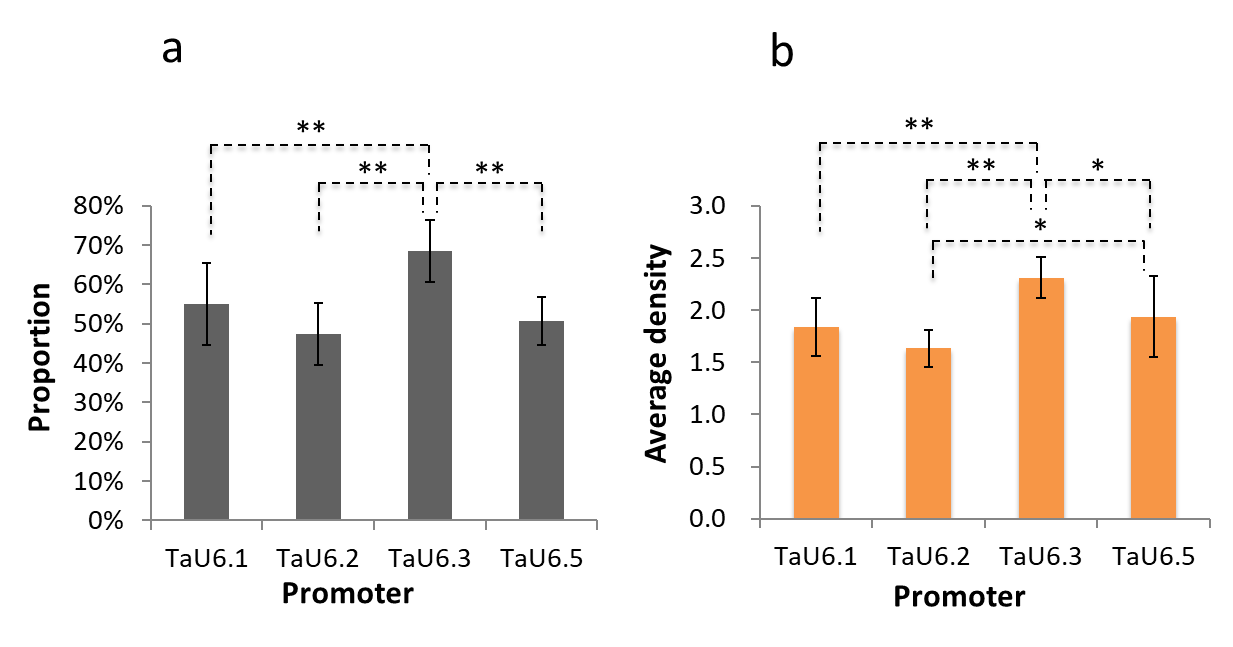
**

**Figure S1.** Comparison in editing efficiency of gGFP driven by different wheat U6 promoters in terms of the proportion of fluorescence cell (a) and average fluorescence intensity per cell (b). Promoters are indicated in the x-axis. The numbers in the y-axis indicate the proportion of protoplasts with GFP relative to the total number of protoplasts (a) and the average density of GFP signal in individual protoplasts (b). The error bars indicate the standard deviation of the mean calculated from five biological replicates. * and ** indicate the significant difference at p< 0.05 and *P* < 0.01 examined by the student’s t-test, respectively.


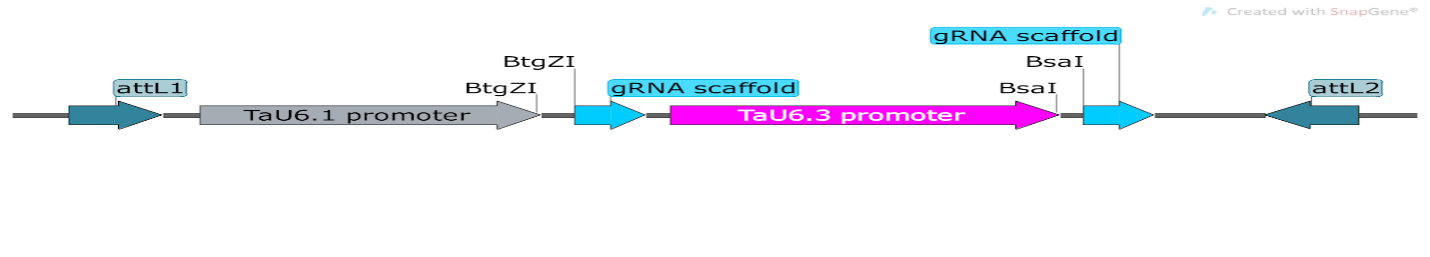


**Figure S2.** Structure of pTagRNA4 in the pENTR4 backbone. Two sgRNAs are inserted into *Btg*ZI-*Btg*ZI and *Bsa*I-*Bsa*I sites sequentially.


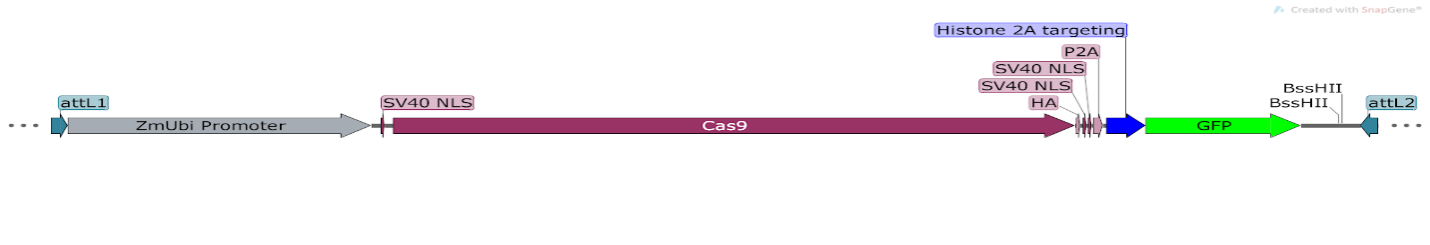


**Figure S3.** Structure of pCas9-GFP used in the transgene. pTaU6-gRNA cassette is inserted into *Bss*HII sites through Gibson assembly.


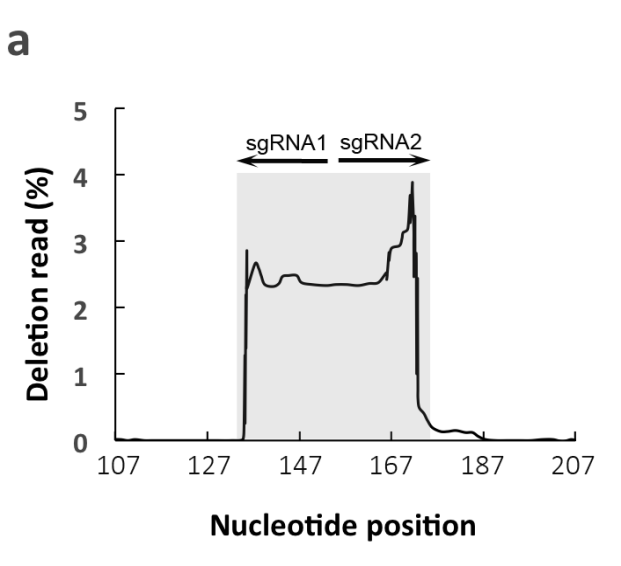


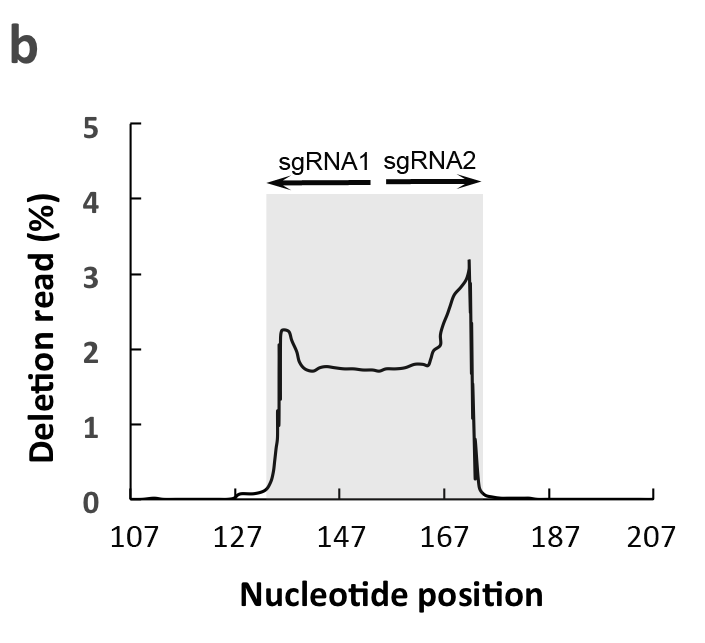


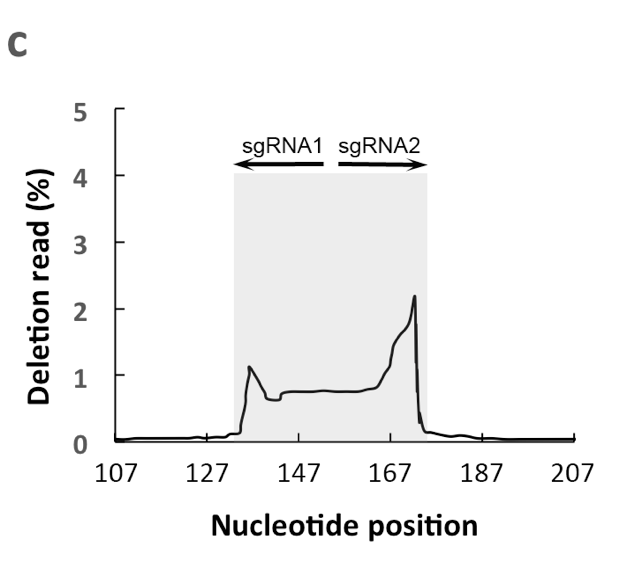


**Figure S4.** Deletion distribution along the amplicon in *TaCKX2-A1* (a), *TaCKX2-B1* (b), and *TaCKX2-D1* (c). The number on X-axis indicates the nucleotide position at the amplicon.  Shaded indicates the sgRNA target region. Arrowed lines denote the orientation of the sgRNA.


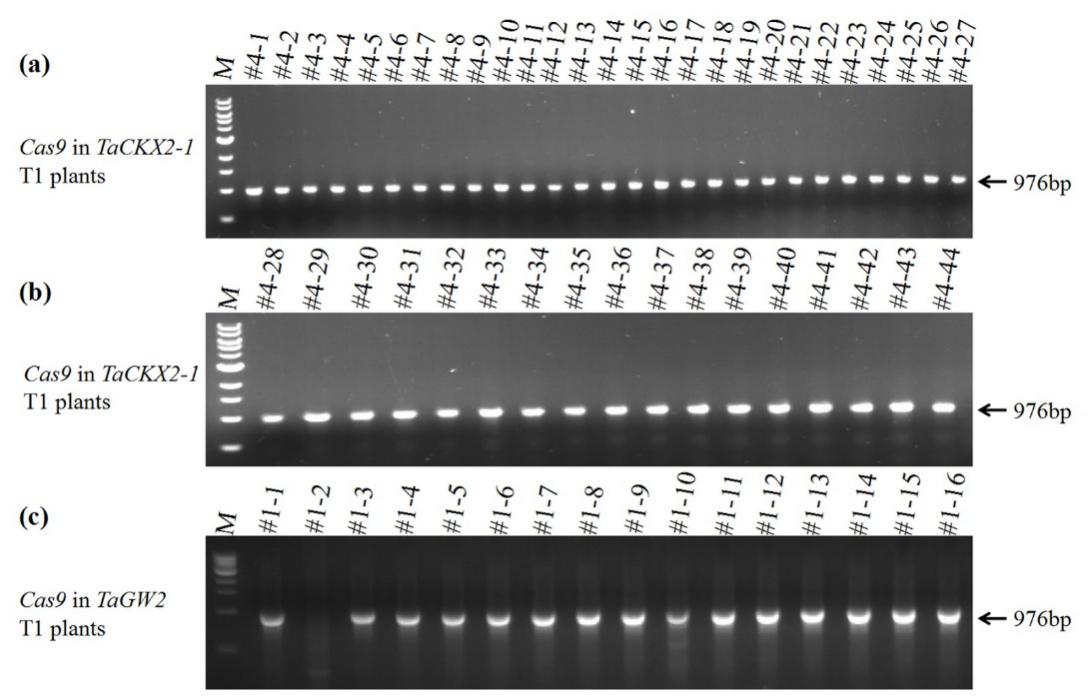


**Figure S5.** Identification of *Cas9*-positive transgenic plants in T_1_ progenies. (a) and (b) Identification of *Cas9* gene in transgenic *TaCKX2-1* #4 T_1_ plants. (c) Identification of *Cas9* gene in transgenic *TaGW2* #1 T_1_ plants. The predicted PCR product size was indicated with black arrows.


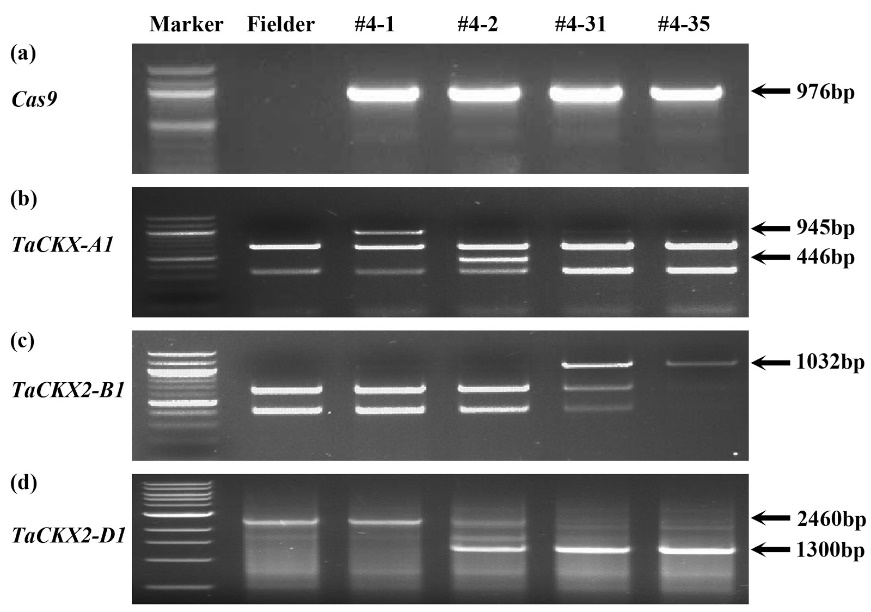


**Figure S6.**  Targeted *TaCKX2-1* homoeologues using the CRISPR/Cas9 system in transgenic T_1_ plants. (a) Identification of *Cas9* gene in transgenic T_1_ plants. The “Fielder” was used as negative control, Lanes #4-1, #4-2, #4-31 and #4-35 represent independent transgenic T_1_ plants. (b) Detection of the mutation in the *TaCKX2-A1* loci by using *Xcm*I digestion. (c) Detection of the mutation in the *TaCKX2-B1* loci by using *Xcm*I digestion. (d) Detection of the large deletion in the *TaCKX2-D1* loci by using gene specific primer amplification. The predicted PCR products size were indicated with black arrows.


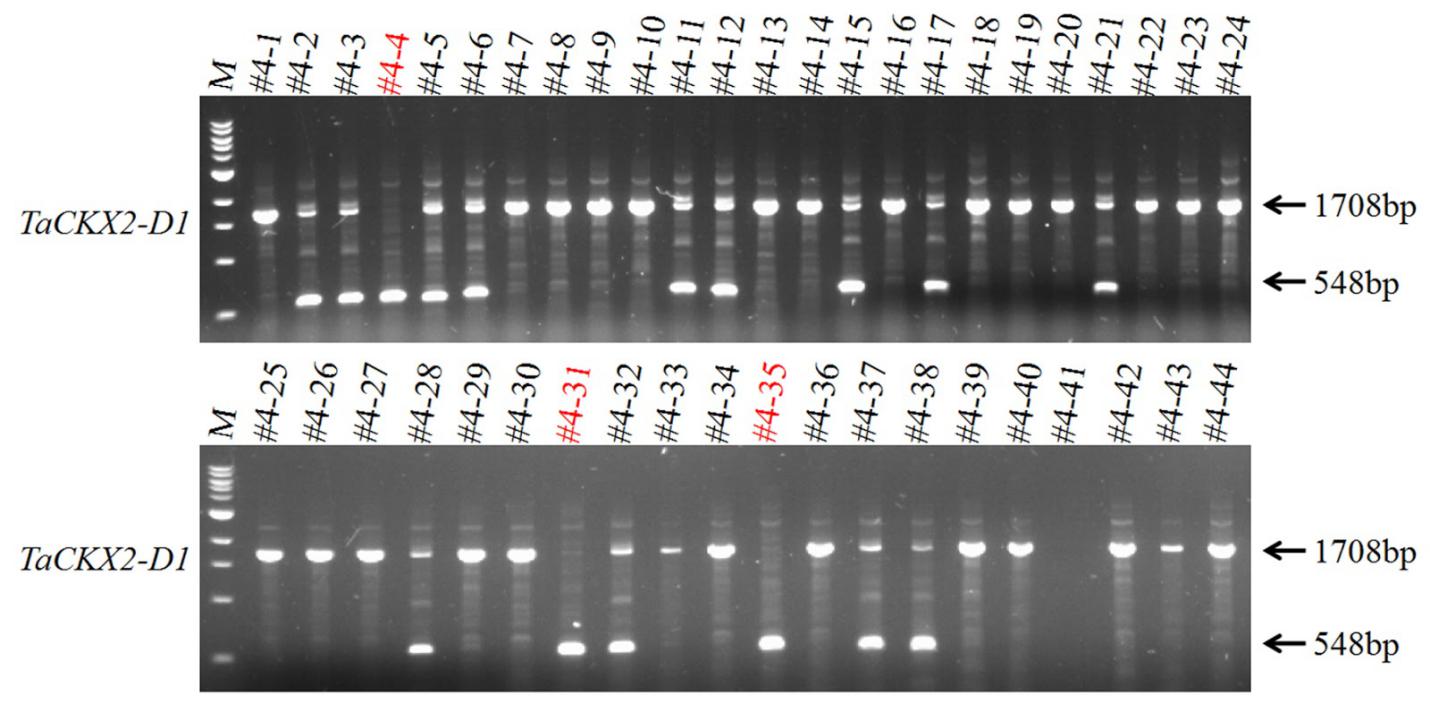


**Figure S7.**  Screen mutant plants in *TaCKX2-D1* # 4 T_1_ plants using PCR assay.  The predicted PCR products size were indicated with black arrows. The numbers in red represent homozygous mutant plants.

**
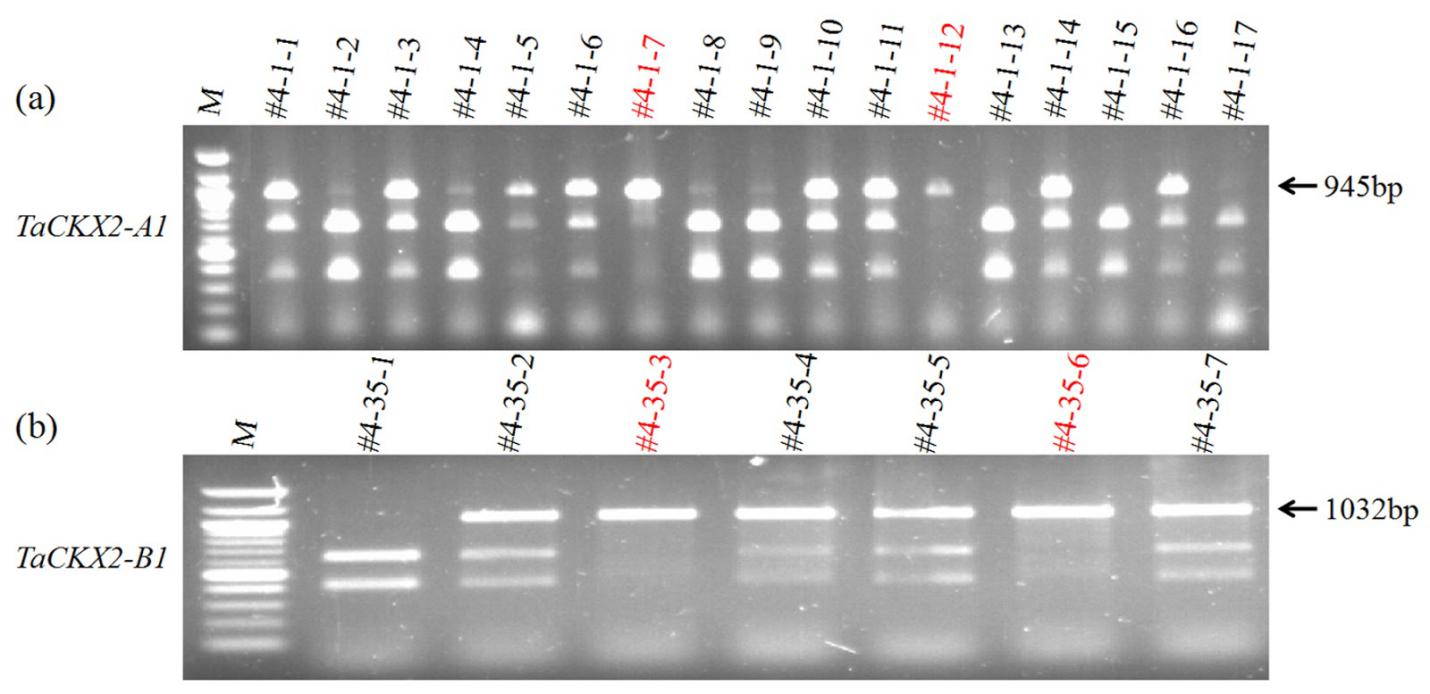
**

**Figure S8.**  Screen mutations in *TaCKX2-1* of plants from the # 4-1 and # 4-35 T_2_ family by the PCR-RE assay. (a) Detection of the genotype in the *TaCKX2-A1* loci of the T_2_ progeny of # 4-1 family by using *Xcm*I digestion. (b) Detection of the genotype in the *TaCKX2-B1* loci of the T2 progeny of # 4-35 family by using *Xcm*I digestion. The predicted PCR products size were indicated with black arrows at the left of the figures. The numbers in red represent homozygous mutant plants.


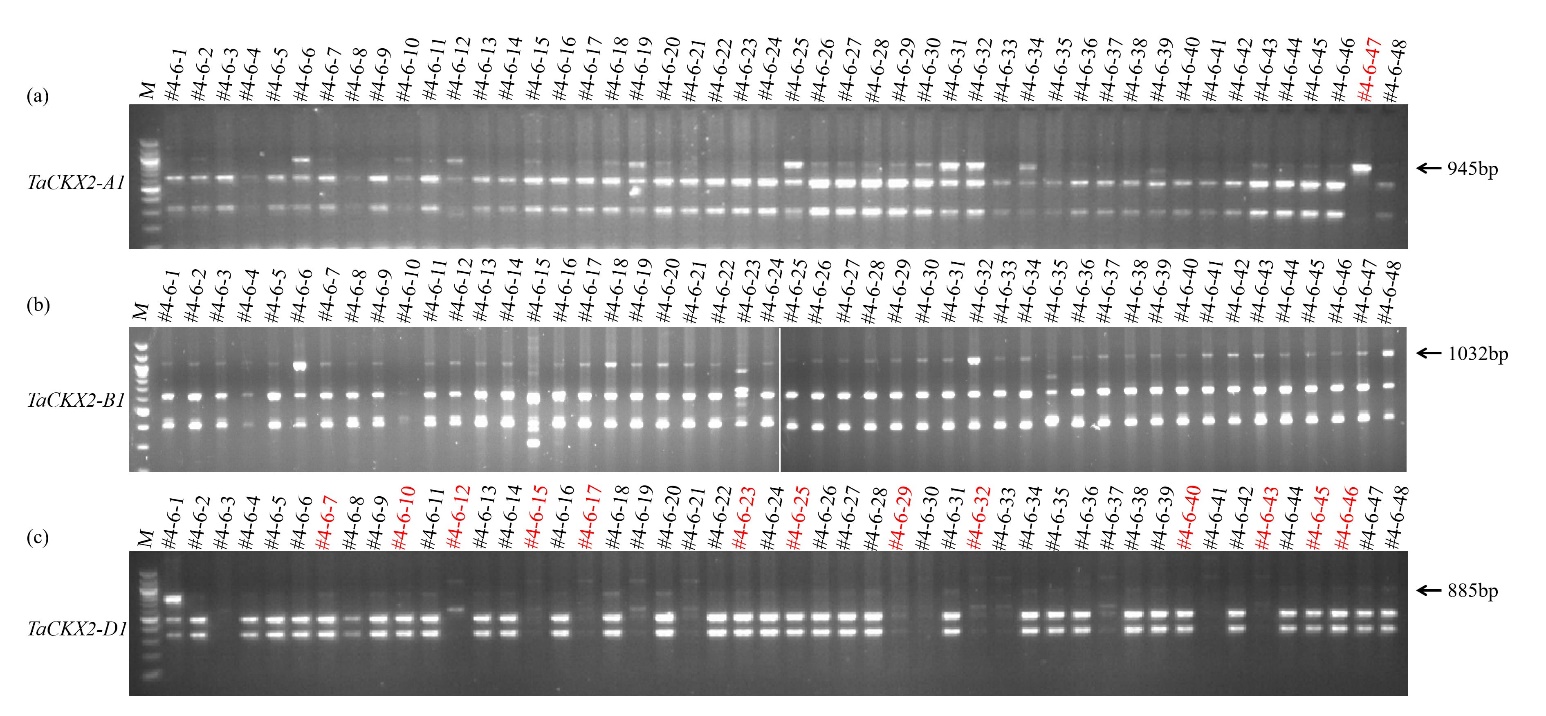


**Figure S9.**  Screen mutations in *TaCKX2-1* of the T_2_ plants from the # 4-6 family by the PCR-RE assay. (a) Detection of the genotype in the *TaCKX2-A1* loci of the T_2_ progeny by using *Xcm*I digestion. (b) Detection of the genotype in the *TaCKX2-B1* loci of the T_2_ progeny by using *Xcm*I digestion. (c) Detection of the genotype in the *TaCKX2-D1* loci of the T_2_ progeny by using *Bgl*I digestion. The predicted PCR products size were indicated with black arrows at the left of the figures. The numbers in red represent homozygous mutant plants.


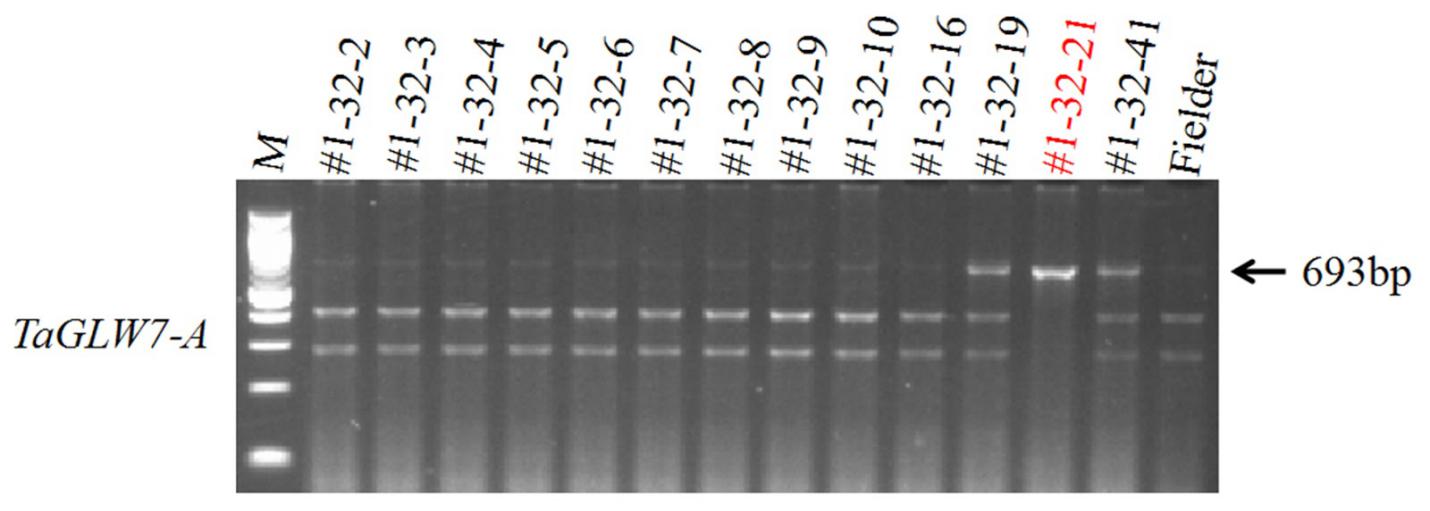


**Figure S10.**  Screening of mutations in *TaGLW7* of the T_2_ plants from the # 1-32 family by the PCR-RNP assay. The wild type PCR products were indicated with the black arrow at the left of the figure. The number in red represents homozygous mutant plant.


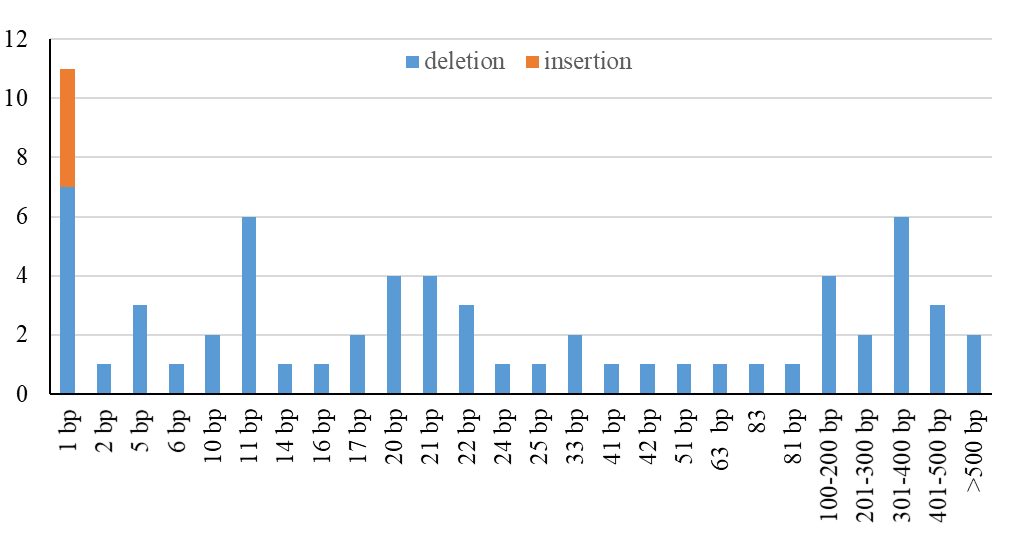


**Figure S11.** Types and spectrum of 68 edit mutations. The numbers in the x axis indicate the size of deletions (blue bars) and insertions (orange bar). The numbers in the y axis indicate the number of mutations.
