# Supplementary material for: Development of an Agrobacterium‐delivered CRISPR/Cas9 system for wheat genome editing
Source: Plant Biotechnol J. 2019 Mar 12;17(8):1623–35. doi: 10.1111/pbi.13088 (PMC6662106; doi:10.1111/pbi.13088)
Supplement: Supplementary file 2 — Table S1 sgRNA target sites used in this study. Table S2 Verification of transgenes in T0 plants. Table S3 Frequencies of CRISPR/Cas9‐induced novel mutations in T0, T1, and T2 populations. Table S4 Types of CRISPR/Cas9‐induced mutations for target genes in T0, T1, and T2 generations. Table S5 Genotypes of mutant plants induced by CRISPR/Cas9 system in T1 population. Table S6 Genotypes of mutant plants induced by CRISPR/Cas9 system in TaCKX2 T2 family #4‐6. Table S7 A summary of the double and triple mutants recovered in TaCKX2‐1. Table S8 Off‐targeting of designed sgRNA for TaCKX2‐1 gene. Table S9 PCR primers used in this study. [file PBI-17-1623-s003.docx]

**Table S1. sgRNA target sites used in this study**

| Gene Name | sgRNA name | Target sequence 5’ to 3’ |
| --- | --- | --- |
| *TaCKX2-1* | sg-TaCKX2-1-1 | AACGCCACCCTGGCGGCCTC |
|  | sg-TaCKX2-1-2 | GGACTTCGGCAACATCACGG |
| *TaGW2* | sg-TaGW2-1 | GAAGAAGCTACGCAAGTTG |
|  | sg-TaGW2-2 | CGAGGCCAAGCTCGCGCCC |
| *TaGLW7* | sg-TaGLW7-1 | TCTCTTCTGTCAAGCAGCC |
|  | sg-TaGLW7-2 | CTCTTCTGTCATGCAGCCAT |
| *TaGW8* | sg-TaGW8-1 | GGCCGGGGATGACAGAAGA |
|  | sg-TaGW8-2 | CAGAAGAGAGAGAGCACAGT |

**Table S2. Verification of transgenes in T**_0_ **plants***

| Target gene | Line No. | Cas9 | sgRNA | Target gene | Line No. | Cas9w | sgRNA |
| --- | --- | --- | --- | --- | --- | --- | --- |
| *TaCKX2-1* | 1 | Y | Y | *TaGW2* | 4 | Y | Y |
| *TaCKX2-1* | 2 | Y | Y | *TaGLW7* | 1 | Y | Y |
| *TaCKX2-1* | 3 | Y | Y | *TaGLW7* | 2 | Y | Y |
| *TaCKX2-1* | 4 | Y | Y | *TaGW8* | 1 | Y | Y |
| *TaCKX2-1* | 5 | N | N | *TaGW8* | 2 | Y | Y |
| *TaCKX2-1* | 6 | Y | Y | *TaGW8* | 3 | Y | Y |
| *TaCKX2-1* | 7 | Y | Y | *TaGW8* | 4 | Y | Y |
| *TaCKX2-1* | 8 | Y | Y | *TaGW8* | 5 | Y | Y |
| *TaGW2* | 1 | Y | Y | *TaGW8* | 6 | Y | Y |
| *TaGW2* | 2 | Y | Y | *TaGW8* | 7 | Y | Y |
| *TaGW2* | 3 | Y | Y | *TaGW8* | 8 | Y | Y |

*Y: the existence of Cas9 or sgRNA; N: absence of Cas9 or sgRNA.

**Table S3. Frequencies of CRISPR/Cas9-induced novel mutations in T_0_, T_1_, and T_2_ populations**

| **Target gene** | **T_0_ generation** | | **T_1_ generation** | | **T_2_ generation** | |
| --- | --- | --- | --- | --- | --- | --- |
|  | Total number of plants | Number of mutated plants | Total number of plants | Number of mutated plants | Total number of plants | Number of mutated plants |
| *TaCKX2-A1*  *TaCKX2-B1*  *TaCKX2-D1* | 8 | 0 | 44 | 2 | 231  (180)* | 27 (27)* |
|  |  | 0 |  | 2 |  | 19 (18)* |
|  |  | 1 |  | 0 |  | 8 (6)* |
| *TaGW2-A*  *TaGW2-B*  *TaGW2-D* | 4 | 0 | 15 | 1 | 93 | 1 |
|  |  | 0 |  | 0 |  | 0 |
|  |  | 0 |  | 1 |  | 0 |
| *TaGLW7-A*  *TaGLW7-B*  *TaGLW7-D* | 2 | 0 | 74 | 2 | 90 | 2 |
|  |  | 0 |  | 0 |  | 0 |
|  |  | 0 |  | 0 |  | 1 |
| *TaGW8-A*  *TaGW8-B*  *TaGW8-D* | 8 | 0 | 93 | 1 | ND | ND |
|  |  | 0 |  | 0 |  | ND |
|  |  | 0 |  | 1 |  | ND |

*The numbers in parentheses are from the T_2_ family #4-6.

**Table S4. Types of CRISPR/Cas9-induced mutations for target genes in T_0_, T**_1_, **and T**_2_ **generations**

| Target gene | Mutation type | Plants with the mutation |
| --- | --- | --- |
| *TaCKX2-A1* | First site: 1-bp deletion | 4-6-176, 4-6-226 |
|  | First site: 2-bp deletion | 4-6-47 |
|  | First site: 6-bp deletion | 4-6-67 |
|  | First site: 11-bp deletion | 4-6-25, 4-6-32 |
|  | First site:14-bp deletion | 4-6-59 |
|  | First site: 20-bp deletion | 4-6-95, 4-6-155, 4-6-175, 4-6-205 |
|  | First site: 21-bp deletion | 4-6-31, 4-6-146 |
|  | First site: 24-bp deletion and 1-bp substitution | 4-1 |
|  | First site: 499-bp deletion | 4-2 |
|  | First site: 1-bp insertion | 4-6-6, 4-6-132 |
|  | Second site: 1-bp insertion | 4-6-110 |
|  | Both sites: 33-bp deletion | 4-6-157 |
|  | Both sites: 33-bp deletion | 4-6-187 |
|  | Both sites: 41-bp deletion | 4-6-166 |
|  | Both sites: 63-bp deletion | 4-6-19 |
|  | Both sites: 164-bp deletion | 4-6-212, 4-6-223 |
|  | Both sites: 81-bp deletion | 4-6-34 |
|  | Both sites: 213-bp deletion | 4-6-171 |
|  | Both sites: 403-bp deletion and 2-bp substitution | 4-6-60 |
|  | Both sites: 459-bp deletion | 4-6-76 |
|  | Both sites: 671-bp deletion | 4-6-229 |
| *TaCKX2-B1* | First site: 1-bp deletion | 4-35, 4-7-88, 4-6-144 |
|  | First site: 1-bp deletion and 24-bp substitution | 4-6-212 |
|  | First site: 11-bp deletion | 4-31, 4-6-32, 4-6-65 |
|  | First site: 20-bp deletion | 4-6-157 |
|  | First site: 21-bp deletion | 4-6-129 |
|  | First site: 22-bp deletion | 4-6-6 |
|  | First site: 33-bp deletion | 4-6-48 |
|  | Second site: 5-bp deletion | 4-6-9, 4-6-162 |
|  | Second site: 22-bp deletion | 4-6-34 |
|  | Both site: 51-bp deletion | 4-6-151 |
|  | Both sites: 106-bp deletion, 2-bp substitution, and 38-bp deletion | 4-6-15 |
|  | Both sites: 138-bp deletion | 4-6-204 |
|  | Both sites: 379-bp deletion and 2-bp substitution | 4-6-217 |
|  | Both sites: 327-bp deletion | 4-6-62 |
|  | Both sites: 372-bp deletion | 4-6-23 |
|  | First site:11-bp deletion | 4-6-231 |
| *TaCKX2-D1* | First site:17-bp deletion | 4-6-93 |
|  | First site:21-bp deletion | 4-6-228 |
|  | Both sites: 42-bp deletion | 4-6-1 |
|  | Both sites: 249-bp deletion | 4-6-155 |
|  | Both sites: 360-bp deletion | 4-30-6 |
|  | Both sites: 373-bp deletion | 4-1-9 |
|  | Both sites: 400-bp deletion | 4-6-121 |
|  | Both sites: 880-bp, 280-bp deletion and 1-bp substitution | 4 (T_0_) |
|  | First site: 22-bp deletion | 1-12-26 |
| *TaGW2-A* | Both sites: 17-bp deletion | 1-14 |
|  | First site: 1-bp deletion | 1-12 |
| *TaGW2-D* | First site: 10-bp deletion | 1-38 |
| *TaGLW7-A* | First site: 25-bp deletion | 1-32 |
|  | First site: 1-bp insertion | 1-32-23, 1-32-37 |
|  | First site: 5-bp deletion | 1-32-10 |
| *TaGLW7-D* | Both sites: 83-bp deletion | 1-93 |
| *TaGW8-A* | First site: 16-bp deletion | 1-73 |
| *TaGW8-D* |  |  |

**Table S5. Genotypes of mutant plants induced by CRISPR/Cas9 system in T**_1_ **population***

| Plant ID | Genotype of *TaCKX2-A1* | Genotype of *TaCKX2-B1* | Genotype of *TaCKX2-D1* |
| --- | --- | --- | --- |
| T_1_-4-1 | Aa (-24 bp) | BB | DD |
| T_1_-4-2 | Aa (-499 bp) | BB | Dd (-1,160 bp)** |
| T_1_-4-3 | AA | BB | Dd (-1,160 bp)** |
| T_1_-4-4 | AA | BB | dd (-1,160 bp)** |
| T_1_-4-5 | AA | BB | Dd (-1,160 bp)** |
| T_1_-4-6 | AA | BB | Dd (-1,160 bp)** |
| T_1_-4-11 | AA | BB | Dd (-1,160 bp)** |
| T_1_-4-12 | AA | BB | Dd (-1,160 bp)** |
| T_1_-4-15 | AA | BB | Dd (-1,160 bp)** |
| T_1_-4-17 | AA | BB | Dd (-1,160 bp)** |
| T_1_-4-21 | AA | BB | Dd (-1,160 bp)** |
| T_1_-4-28 | AA | BB | Dd (-1,160 bp)** |
| T_1_-4-31 | AA | Bb (-11 bp) | dd (-1,16 0bp) |
| T_1_-4-32 | AA | BB | Dd (-1,160  bp) |
| T_1_-4-35 | AA | Bb (-1 bp) | dd (-1,160 bp)** |
| T_1_-4-37 | AA | BB | Dd (-1,160 bp)** |
| T_1_-4-38 | AA | BB | Dd (-1,160 bp)** |
|  | | | |
| Plant ID | Genotype of *TaGW2-A* | Genotype of *TaGW2-B* | Genotype of *TaGW2-D* |
| T_1_-1-12 | AA | BB | Dd (-17 bp) |
| T_1_-1-14 | Aa (-1 bp) | BB | DD |
|  | | | |
| Plant ID | Genotype of *TaGLW7-A* | Genotype of *TaGLW7-B* | Genotype of *TaGLW7-D* |
| T_1_-1-32 | Aa (-25 bp) | BB | DD |
| T_1_-1-38 | Aa (-10 bp) | BB | DD |
|  | | | |
| Plant ID | Genotype of *TaGW8-A* | Genotype of *TaGW8-B* | Genotype of *TaGW8-D* |
| T_1_-1-73  T_1_-1-93 | AA  Aa (-83 bp) | BB  BB | Dd (-16 bp)  DD |

*Homozygous wild type genotypes are expressed as AA, BB, and DD for the A, B, and D genome copies, respectively; homozygous mutant alleles as aa, bb, and dd; and the heterozygous genotypes as Aa, Bb, and Dd. The mutation types are included in the parentheses, and “-“ indicates a deletion and “+” an insertion.

**The 1,160-bp deletion is compound mutation containing an 880-bp deletion, a 280-bp deletion, and a 1-bp substitution.

**Table S6. Genotypes of mutant plants induced by CRISPR/Cas9 system in *TaCKX2* T2 family #4-6***

| Plant ID | Genotype of *TaCKX2-A1* | Genotype of *TaCKX2-B1* | Genotype of *TaCKX2-D1* |
| --- | --- | --- | --- |
| T_2_-4-6-1 | AA | BB | Dd (- 42 bp) [1, 2] |
| T_2_-4-6-6 | Aa (+ 1 bp) [1] | Bb (- 21 bp) [1] | Dd (- 1,160 bp) [1, 2]*** |
| T_2_-4-6-9 | AA | Bb (- 5 bp) [2] | DD |
| T_2_-4-6-15 | AA | Bb (- 106 bp, -38 bp, 2 bp sub) [1, 2] | dd (- 1,160 bp) [1, 2]*** |
| T_2_-4-6-19 | Aa (- 63 bp ) [1, 2] | BB | Dd (- 1,160 bp) [1, 2]*** |
| T_2_-4-6-23 | AA | Bb (-372 bp) [1, 2] | dd (- 1,160 bp) [1, 2]*** |
| T_2_-4-6-25 | Aa (- 11 bp) [1] | BB | dd (- 1,160 bp) [1, 2]*** |
| T_2_-4-6-31 | Aa (- 21 bp) [1] | BB | DD |
| T_2_-4-6-32 | Aa (- 11 bp) [1] | Bb (- 11 bp) [1] | dd (- 1,160 bp) [1, 2]*** |
| T_2_-4-6-34 | Aa (- 81 bp) [2] | Bb (- 22 bp) [2] | DD |
| T_2_-4-6-47 | aa (- 2 bp) [1]** | BB | DD |
| T_2_-4-6-48 | AA | Bb (-33 bp) [1] | Dd (- 1,160 bp) [1, 2]*** |
| T_2_-4-6-59 | Aa (- 14 bp) [1] | BB | DD |
| T_2_-4-6-60 | Aa (- 403 bp, 2-bp sub) [2] | BB | DD |
| T_2_-4-6-62 | AA | Bb (- 327 bp) [1, 2] | DD |
| T_2_-4-6-65 | AA | Bb (- 11 bp) [1] | DD |
| T_2_-4-6-67 | Aa (- 6 bp) [1] | BB | DD |
| T_2_-4-6-76 | Aa (- 459 bp) [2] | BB | DD |
| T_2_-4-6-93 | AA | BB | Dd (- 17 bp) [1] |
| T_2_-4-6-95 | Aa (-20 bp) [1] | BB | DD |
| T_2_-4-6-110 | Aa (- 1 bp) [2] | BB | DD |
| T_2_-4-6-121 | AA | BB | Dd (-400 bp) |
| T_2_-4-6-129 | AA | Bb (-21 bp) [1] | dd (- 1,160 bp) [1, 2]*** |
| T_2_-4-6-132 | Aa (+ 1) [1] | BB | DD |
| T_2_-4-6-144 | AA | Bb (- 1 bp) [1] | DD |
| T_2_-4-6-146 | Aa (- 21 bp) [1] | BB | DD |
| T_2_-4-6-151 | AA | Bb (- 51 bp) [2] | dd (- 1,160 bp) [1, 2]*** |
| T_2_-4-6-155 | Aa (-20 bp) [1] | BB | Dd (- 249 bp) [1, 2] |
| T_2_-4-6-157 | Aa -33 bp) [1] | Bb (- 20 bp) [1] | Dd (- 1,160 bp) [1, 2]*** |
| T_2_-4-6-162 | AA | Bb (- 5 bp) [2] | DD |
| T_2_-4-6-166 | Aa (- 41 bp) [1, 2] | BB | DD |
| T_2_-4-6-171 | Aa (- 213 bp) [2] | BB | DD |
| T_2_-4-6-175 | Aa (- 20 bp) [1] | BB | DD |
| T_2_-4-6-176 | Aa (- 1 bp) [1] | BB | DD |
| T_2_-4-6-187 | Aa (-33 bp) [1, 2] | BB | DD |
| T_2_-4-6-200 | AA | Bb ( 2 bp sub) [1, 2] | dd (- 1,160 bp) [1, 2]*** |
| T_2_-4-6-205 | Aa (- 20 bp ) [1] | BB | Dd (- 1,160 bp) [1, 2]*** |
| T_2_-4-6-212 | Aa (- 164 bp) [1, 2] | Bb (-10 bp) [1] | dd (- 1,160 bp) [1, 2]*** |
| T_2_-4-6-217 | AA | Bb (-200 bp) | DD |
| T2-4-6-223 | Aa (-164 bp) [1, 2] | BB | dd (- 1,160 bp) [1, 2]*** |
| T_2_-4-6-226 | aa (- 1 bp) [1] ** | BB | dd (- 1,160 bp) [1, 2]*** |
| T_2_-4-6-228 | AA | BB | Dd( - 21 bp) [1] |
| T_2_-4-6-229 | Aa (-671 bp) [1, 2] | BB | dd (- 1,160 bp) [1, 2]*** |
| T_2_-4-6-231 | AA | BB | Dd( - 11 bp) [1] |

*Homozygous wild type genotypes are expressed as AA, BB, and DD for the A, B, and D genome copies, respectively; homozygous mutant alleles as aa, bb, and dd; and the heterozygous genotypes as Aa, Bb, and Dd. The mutation types are included in the parentheses, and “-“ indicates a deletion and “+” an insertion.

** Homozygosity due to fertilization of eggs and pollens carrying independent mutations.

*** Mutation of the 1,160-bp deletion (an 880-bp deletion, a 280-bp deletion, and a 1-bp substitution) was inherited from the T_0_ plant #4.

**** Mutations based on PCR product and RE

| **Table S7**. A summary of the double and triple mutants recovered in *TaCKX2-1**   \| Genotypes \| T2 \| T3 \| \| --- \| --- \| --- \| \| AAbbdd \| 0 \| 24 \| \| aaBBdd \| 1 \| 9 \| \| aabbDD \| 0 \| 1 \| \| AAbbdd \| 0 \| 5 \| \| aaBbdd \| 0 \| 5 \| \| AabbDd \| 0 \| 2 \| \| aaBbDd \| 0 \| 2 \| \| AaBbdd \| 1 \| 7 \| \| AaBbDd \| 3 \| 8 \| |
| --- | --- | --- | --- | --- | --- | --- | --- | --- | --- | --- | --- | --- | --- | --- | --- | --- | --- | --- | --- | --- | --- | --- | --- | --- | --- | --- | --- | --- | --- | --- |

*The lower case letters indicate mutation alleles in homoeologous genes in the genomes

Table S8. Off-targeting of designed sgRNA for *TaCKX2-1* gene

| Off-target | Genomic location | Off-target region* | Primer sequence (5’ to 3’) | Mutations |
| --- | --- | --- | --- | --- |
| 1 | 2A:295112146-295112168 | cctAGAGCCACCCTGGCGGCCTC | F: GTGGGCACTAGCTGCTTTCT  R: GGGAGCTTCGGACGTATCATC | No |
| 2 | 4A:164487356-164487378 | ctgAACGCCACCCTGGCGGCTGG | F: GGTTTGTGGCAGGATTTCTT  R: CGGCTTGCAACTTGGTGACT | No |
| 3 | 2A:193623044-193623066 | cctGGCGCCACCCTGGCGGCCTC | F: CATACGTGCATACATACGAC  R: GGCTTGCTTCTAGCTGCTTCC | No |
| 4 | 4A:479502495-479502517 | cctAGCGCCACCCTGGCGGCCTT | F: TCTTTCCCCTTAGCTTCAGCCA  R: CATCAAGAGGCTCACTGGA | No |

*The positions mismatching with the preselected target are shaded in yellow, and the PAM motifs are in lower case and shaded in green.

**Table S9. PCR primers used in this study**

| Primer name | Primer sequence (5' to 3') | Application |
| --- | --- | --- |
| *TaCKX2-A1*-F  *TaCKX2-A1*-R | CAACGCACGGACACTTAGC  CGTGTGCGTACGTAGAAGATGAG | To amplify the *TaCKX2-A1* first exon region |
| *TaCKX2-B1*-F  *TaCKX2-B1*-R | TCCTGTGCTGTGTACCAGAG  ATGAACGTGATTCCACGCC | To amplify the *TaCKX2-B1* first exon region |
| *TaCKX2-D1*-1F  *TaCKX2-D1*-1R | CATTTCATTTATCATGACTTCCC  GTGCGTGCTTGCCCATATC | To amplify the *TaCKX2-D1* first exon region |
| *TaGW2-A*-F  *TaGW2-A*-R | CGTGTCACAAAACTAATTGGG  CTACGGCAGAACAAATGCAA | To amplify the *TaGW2-A* first exon region |
| *TaGW2-B*-F  *TaGW2-B*-R | GGGCCAGCAGCAGAGAGA  CAAACACAGGCACCTAGCAC | To amplify the *TaGW2-B* first exon region |
| *TaGW2-D*-F  *TaGW2-D*-R | GACATCATACAAGTGGGGAAGGA  TACGGCAGAACAAATGCAAC | To amplify the *TaGW2-D* first exon region |
| *TaGLW7-A*-F  *TaGLW7-A*-R | TATCAATGTTGTGCCTGTTTCT  CCAAGTGAAAACCAAATGCTC | To amplify the *TaGLW7-A* 3’ UTR region |
| *TaGLW7-B*-F  *TaGLW7-B*-R | GCAAGATAAAAACTGGAGG  GGCGATAAGGTTTATAAGG | To amplify the *TaGLW7-B* 3’ UTR region |
| *TaGLW7-D*-F  *TaGLW7-D*-R | TTAATTCGCTCCAGTTAAGCA  GGCCCGTTCTGAGCAATC | To amplify the *TaGLW7-D* 3’ UTR region |
| *TaGW8-A*-F  *TaGW8-A*-R | CTCGCATCATAAGAATGGAAG  TGGCAGTTCATCTCGTTGTC | To amplify the *TaGW8-A* third exon region |
| *TaGW8-B*-F  *TaGW8-B*-R | AAATTCAGGGACAAGGTTCG  TGCGGTTATTCATTGCTTTC | To amplify the *TaGW8-B*  third exon region |
| *TaGW8-D*-F  *TaGW8-D*-R | GACGTACTGTTCTTCAGTAGCC  TCATCTCGTTGTCGTTGGAG | To amplify the *TaGW8-D* third region |
| *Cas9*-F  *Cas9*-R | GGGCCCGGTAGTTCTACTTC  ATTTCTTCGATGGCACCTTG | To amplify the *Cas9* gene for transgenic plant confirmation |
| T7-sg-TaGW2-1-F  T7-sg-TaGW2-1-R | ATAGCAACTTGCGTAGCTTCTTC  AAACGAAGAAGCTACGCAAGTTG | To construct vector for *in vit*ro sgRNA transcription |
| T7-sg-TaGW2-2-F  T7-sg-TaGW2-2-R | ATAGGGGCGCGAGCTTGGCCTCG  AAACCGAGGCCAAGCTCGCGCCC | To construct a vector for *in vit*ro sgRNA transcription |
| T7-sg-TaGLW7-1-F  T7-sg-TaGLW7-1-R | ATAGTGGCTGCTTGACAGAAGAGA  AAACTCTCTTCTGTCAAGCAGCCA | To construct a vector for *in vit*ro sgRNA transcription |
| T7-sg-TaGLW7-2-F  T7-sg-TaGLW7-2-R | ATAGATGGCTGCATGACAGAAGAG  AAACCTCTTCTGTCATGCAGCCAT | To construct a vector for *in vit*ro sgRNA transcription |
| GFP+1-F    GFPSXK-R | CACCGGATCCACCATGGTGAGCAAGGgGCGAGGAG  GGCCGGTACCTCATCTAGAACTAGTTCCGGACTTGTACAGCTCGTC | To introduce a nucleotide insertion (lower-case letter) into GFP CDS. |
| MiSCKX2-1-F1    MiSCKX2-1-R1 | CTCTTTCCCTACACGACGCTCTTCCGATCTTCGTTTACGTGCTTCTGGTG  CTGGAGTTCAGACGTGTGCTCTTCCGATCTCGCGGAACGAGATGGTGTA | To amplify *TaCKX2-1* from all three homeologs |
| TruSeq-F    TruSeq-R2 | AATGATACGGCGACCACCGAGATCTACACTCTTTCCCTACACGACGCTCT  CAAGCAGAAGACGGCATACGAGATacatcgGTGACTGGAGTTCAGACGTGTGCT | Used for the second round PCR and adding barcode sequence (lower case letters) |
| *TaCKX2-D1*-2F  *TaCKX2-D1*-2R | TCGTTTAGTGGGAGGAGACG  TTTAACGCAGAGGGGAAATG | To amplify the *TaCKX2-D1* large deletion fragment |
| *TaCKX2-D1*-3F  *TaCKX2-D1*-3R | TGACTTTGCCAACCTCAAGA  AACGAGATGGTGTAGGGCC | To amplify the *TaCKX2-D1* large deletion fragment |
