# Supplementary material for: Development of an Agrobacterium‐delivered CRISPR/Cas9 system for wheat genome editing
Source: Plant Biotechnol J. 2019 Mar 12;17(8):1623–35. doi: 10.1111/pbi.13088 (PMC6662106; doi:10.1111/pbi.13088)
Supplement: Supplementary file 3 — Appendix S1 Sequences of U6 promoters. [file PBI-17-1623-s002.docx]

Appendix S1. Sequences of U6 promoters

>TaU6.1

ACCAAGCCCGTTATTCTGACAGTTCTGGTGCTCAACACATTTATATTTATCAAGGAGCACATTGTTACTCACTGCTAGGAGGGAATCGAACTAGGAATATTGATCAGAGGAACTACGAGAGAGCTGAAGATAACTGCCCTCTAGCTCTCACTGATCTGGGTCGCATAGTGAGATGCAGCCCACGTGAGTTCAGCAACGGTCTAGCGCTGGGCTTTTAGGCCCGCATGATCGGGCTTTTGTCGGGTGGTCGACGTGTTCACGATTGGGGAGAGCAACGCAGCAGTTCCTCTTAGTTTAGTCCCACCTCGCCTGTCCAGCAGAGTTCTGACCGGTTTATAAACTCGCTTGCTGCATCAGACTTG

>TaU6.2

GTTGGGGCTAGATTGCTGCCAGCAGCTAGAGAATACTCTGGGACAACTGAACACAACACAAGCTAACTCTGGGAACAAACTCAACACGATGATGTTCATGTTCGGCGTGTGGCAAAGTACACGAGTAGGACGGGGAGGAAGAAAAAACAGCACTATGCTCTGTCTCTCTACCGATTTGGGCCATGTAATGAGATGCAGCCCACGTGAGGTCTACTGCGAGGCTGCTTGCTGCTGGGCTTTTAGGGCCCAGGATGGCTTACGCGCTCGCGGGAGCGGCCGGGGGAGCAGAACACAGCAGTCTAGGATTGTTTAGTCCCACCTCGCCTACGCGAGGCCGTTTCGACCGATTTATAAGCTCGGTCGCTGCATCAGACAC

>TaU6.3

ACTCTGCGAGGACTTCTCTTGTGAAAAGCTCGAAATAAATCTACATACACGTTTATGTTAAAATGTGAGAAGAATGTAGAATGACTAGTATAAAAACGGAGTCAAATAACCAGCGTTCTCCCCCACGGTGAAAAAAGAACCTATCACATCGGTAGACCATTTTTTTATAGGCAAATCATATCGGCAGACTAACAGGAAGGCTCACTATTCCGTACTAGCACGATGGCCCATTACGGTCTTCCACTATTTTGGGCCACACCGAGAGATCCAGCCTACTCGTAGCCCACCGCACGTTCCGACGGCGCTTCCGTCTGGACAAGCAACGGGGGGAGCAGAAGGCTCGTTTGTTTAGTCCCACATCGGCTAGCGAAAGGGAGAACGACCAGTTTATAAGCCACGCGCTGCACACAGACTC

>TaU6.5

GGTTTGCACTAGCTGCTTCCCCTGATTTAGCTTGTTGGGTCTACTGTCAGATGCACTAGGCTCCTGCTTTTTCCACAGACGTGTTGGCCTGTTACTCACTTTCTCTTCTGAAAACACACGCCATGACGTGCACGTTTATATTAAAATGCAAGGAGAATGCACAATGGCGAGTATAAAAACGGAGAATGCGCTATGAAGAGAGGATACAGCTGCCTCCTGTGTGGTCTGTTGTTTTACACATCGAGAGAAACAACAAATGAGTTGGTCGCGTTCTCCTTTTTCTTTAAACAACAAAACTAGCCTTCTCCCACACGTTGAAAAAAAAAAAACCCCACCACATCGGGCGACTAGCAGTTCCCCAGAAGCATGAAGGCCCATTACGGTCTTCCATTATTTTGGGCCGCACTAAGAGACCACGCCTACTCGAGGCCCACCGCACGTCCCGATGGCGCTTCCTGCTGGACAAACAACGCGGGGGGCAGAAGGCGTGTTCGTTTAGTCCCACATCGCCTGGCCGAGGAGAGAAGCACCAGTTTATAACCCGCGCGCTGCAGACAGACTC
